# FOXO1‐NCOA4 Axis Contributes to Cisplatin‐Induced Cochlea Spiral Ganglion Neuron Ferroptosis via Ferritinophagy

**DOI:** 10.1002/advs.202402671

**Published:** 2024-08-29

**Authors:** Xue Wang, Lei Xu, Yu Meng, Fang Chen, Jinzhu Zhuang, Man Wang, Weibin An, Yuechen Han, Bo Chu, Renjie Chai, Wenwen Liu, Haibo Wang

**Affiliations:** ^1^ Department of Otolaryngology‐Head and Neck Surgery Shandong Provincial ENT Hospital Shandong University Jinan 250022 China; ^2^ Shandong Institute of Otorhinolaryngology Jinan 250022 China; ^3^ Department of Cell Biology School of Basic Medical Sciences Cheeloo College of Medicine Shandong University Jinan 250012 China; ^4^ State Key Laboratory of Digital Medical Engineering Department of Otolaryngology Head and Neck Surgery Zhongda Hospital School of Life Sciences and Technology School of Medicine Advanced Institute for Life and Health Jiangsu Province High‐Tech Key Laboratory for Bio‐Medical Research Southeast University Nanjing 210096 China; ^5^ Co‐Innovation Center of Neuroregeneration Nantong University Nantong 226001 China; ^6^ Department of Neurology Aerospace Center Hospital School of Life Science Beijing Institute of Technology Beijing 100081 China; ^7^ Department of Otolaryngology Head and Neck Surgery Sichuan Provincial People's Hospital School of Medicine University of Electronic Science and Technology of China Chengdu 610072 China; ^8^ Southeast University Shenzhen Research Institute Shenzhen 518063 China

**Keywords:** cisplatin, ferritinophagy, ferroptosis, forkhead box transcription factor O1, hearing loss, nuclear receptor coactivator 4, spiral ganglion neuron

## Abstract

Mammalian cochlea spiral ganglion neurons (SGNs) are crucial for sound transmission, they can be damaged by chemotherapy drug cisplatin and lead to irreversible sensorineural hearing loss (SNHL), while such damage can also render cochlear implants ineffective. However, the mechanisms underlying cisplatin‐induced SGNs damage and subsequent SNHL are still under debate and there is no currently effective clinical treatment. Here, this study demonstrates that ferroptosis is triggered in SGNs following exposure to cisplatin. Inhibiting ferroptosis protects against cisplatin‐induced SGNs damage and hearing loss, while inducing ferroptosis intensifies these effects. Furthermore, cisplatin prompts nuclear receptor coactivator 4 (NCOA4)‐mediated ferritinophagy in SGNs, while knocking down NCOA4 mitigates cisplatin‐induced ferroptosis and hearing loss. Notably, the upstream regulator of NCOA4 is identified and transcription factor forkhead box O1 (FOXO1) is shown to directly suppress NCOA4 expression in SGNs. The knocking down of FOXO1 amplifies NCOA4‐mediated ferritinophagy, increases ferroptosis and lipid peroxidation, while disrupting the interaction between FOXO1 and NCOA4 in NCOA4 knock out mice prevents the cisplatin‐induced SGN ferroptosis and hearing loss. Collectively, this study highlights the critical role of the FOXO1‐NCOA4 axis in regulating ferritinophagy and ferroptosis in cisplatin‐induced SGNs damage, offering promising therapeutic targets for SNHL mitigation.

## Graphical abstract

This study demonstrates that NCOA4‐mediated ferritinophagy and ferroptosis are induced in spiral ganglion neurons (SGNs) following chemotherapy drug cisplatin treatment. FOXO1 acts as a direct upstream regulator of NCOA4, inhibiting NCOA4‐mediated ferritinophagy and alleviating cisplatin‐induced SGN ferroptosis and hearing loss. These findings confirm the role of the FOXO1‐NCOA4 axis in regulating ferritinophagy and ferroptosis in response to cisplatin‐induced SGN damage, highlighting promising therapeutic targets for mitigating sensorineural hearing loss.

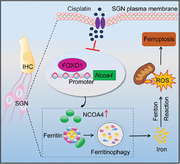

## 1 Introduction

Sensorineural hearing loss (SNHL) is the most common form of hearing impairment globally, significantly affecting the speech and language development of children and causing social and vocational challenges for adults.^[^ ^1^ ^]^ This results in a considerable socioeconomic burden on families and society. SNHL is caused by pathological changes in cochlear hair cells (HCs), auditory nerves, the central auditory system, or a combination of these.^[^ ^2^ ^]^ Spiral ganglion neurons (SGNs), which are primary auditory nerves, are vital for transmitting sound information from cochlear HCs to the auditory cortex.^[^ ^3^ ^]^ Damage to SGNs can lead to irreversible SNHL, as these neurons cannot regenerate once damaged.^[^ ^4^, ^5^ ^]^ The preservation of SGN survival and health is critical for maintaining normal hearing and ensuring the performance of cochlear implants, which are the only effective treatment for individuals with severe to profound SNHL and rely on sufficient functional SGNs to operate effectively.^[^ ^6^ ^]^

Among the multiple factors that cause auditory neuron damage, cisplatin, a broad‐spectrum chemotherapy drug known for its ototoxic effects,^[^ ^7^, ^8^, ^9^ ^]^ can result in primary injury to SGNs, leading to SNHL.^[^ ^10^ ^]^ Currently, there is no effective clinical treatment for cisplatin‐induced SNHL affecting SGNs. Such damage can render cochlear implants ineffective, thus eliminating the sole treatment option for patients with SNHL.^[^ ^11^ ^]^ However, to date, the mechanisms underlying cisplatin‐induced ototoxicity remain under debate. While it is established that cisplatin enhances the production of reactive oxygen species (ROS) in cochlear cells, resulting in oxidative stress and causing cell damage through lipid peroxidation, protein nitration, and apoptosis,^[^ ^12^, ^13^, ^14^ ^]^ the specific mechanisms by which cisplatin damages SGNs, particularly those involving oxidative stress and lipid peroxidation, have yet to be fully elucidated.

Ferroptosis, a form of programmed cell death discovered recently, is iron‐dependent and nonapoptotic, characterized by the accumulation of intracellular iron, elevated levels of ROS, and lipid peroxidation.^[^ ^15^, ^16^, ^17^ ^]^ Cells undergoing ferroptosis exhibit unique mitochondrial changes, but maintain intact cell membranes, normal nuclear sizes, and unchanged chromatin densities.^[^ ^15^ ^]^ Ferroptosis has been associated with pathological cell death observed in various diseases, including neurodegenerative disorders like Alzheimer's, Huntington's, and Parkinson's diseases,^[^ ^18^ ^]^ carcinogenesis,^[^ ^19^ ^]^ stroke,^[^ ^20^ ^]^ kidney degeneration.^[^ ^21^ ^]^ Recent studies have also indicated that ferroptosis contributes to SNHL caused by cochlear HC damage, induced by cisplatin or aminoglycoside antibiotics.^[^ ^22^, ^23^, ^24^, ^25^ ^]^ However, reports on the susceptibility of cochlear SGNs to ferroptosis are still unclear.

Given that iron is essential for the accumulation of lipid peroxides and the execution of ferroptosis, factors regulating iron metabolism significantly influence ferroptosis sensitivity. Within cells, ferritinophagy, a form of selective autophagy, plays a crucial role in modulating ferroptosis sensitivity by controlling iron availability.^[^ ^26^, ^27^, ^28^, ^29^, ^30^ ^]^ The cargo receptor nuclear receptor coactivator 4 (NCOA4) is central to ferritinophagy, as it facilitates the recruitment of ferritin, an iron‐storage protein, for lysosomal degradation and iron release.^[^ ^29^ ^]^ Under normal conditions, a balanced rate of ferritinophagy is essential for maintaining iron homeostasis.^[^ ^31^ ^]^ However, excessive ferritinophagy can lead to an overload of intracellular iron, resulting in the depletion of glutathione (GSH) and a reduction in glutathione peroxidase 4 (GPX4) expression.^[^ ^31^ ^]^ This imbalance promotes lipid peroxidation and cell damage through ROS generated by the Fenton reaction,^[^ ^31^ ^]^ ultimately leading to ferroptosis and resulting in cell death.^[^ ^31^ ^]^ While blocking autophagy significantly mitigated cisplatin‐induced ferroptosis in the HC‐like cell line HEI‐OC1,^[^ ^32^ ^]^ detailed insights into the roles of ferritinophagy and ferroptosis in SGN damage remain scarce.

Forkhead box transcription factor O1 (FOXO1), a member of the FOXO family, is integral in regulating cell proliferation, apoptosis, and oxidative stress and maintaining homeostasis in various diseases.^[^ ^33^ ^]^ In the auditory system, FOXO1 is known to protect against acute acoustic trauma through sound conditioning in rats ^[^ ^34^ ^]^ and to mitigate cisplatin‐induced ototoxicity in vitro.^[^ ^35^ ^]^ However, its involvement in SGN damage remains unverified. Recent findings suggest a link between FOXO1 and ferroptosis in cancer cells and cardiomyocytes,^[^ ^36^, ^37^, ^38^ ^]^ proposing FOXO1 as a crucial regulator of ferroptosis in multifarious conditions. Despite these advancements, research in this area is nascent and calls for more in‐depth investigation.

In this study, we confirmed that ferroptosis was induced in SGNs following exposure to cisplatin. We explored the role of ferroptosis in SGN damage and subsequent hearing loss by utilizing a ferroptosis inducer and an iron chelator. The NCOA4‐mediated ferritinophagy was activated in SGNs after cisplatin treatment, while silencing NCOA4 mitigated the SGN loss, ferroptosis, and improved hearing impairment caused by cisplatin. Additionally, we investigated the effect of the FOXO1‐NCOA4 axis on ferritinophagy and ferroptosis in the context of cisplatin‐induced SGN damage. Our findings point to potential therapeutic targets for the alleviation of SNHL.

## 2 Results

### 2.1 Cisplatin Induces Ferroptosis in Cochlear SGNs both In Vitro and In Vivo

**Figure 1:**
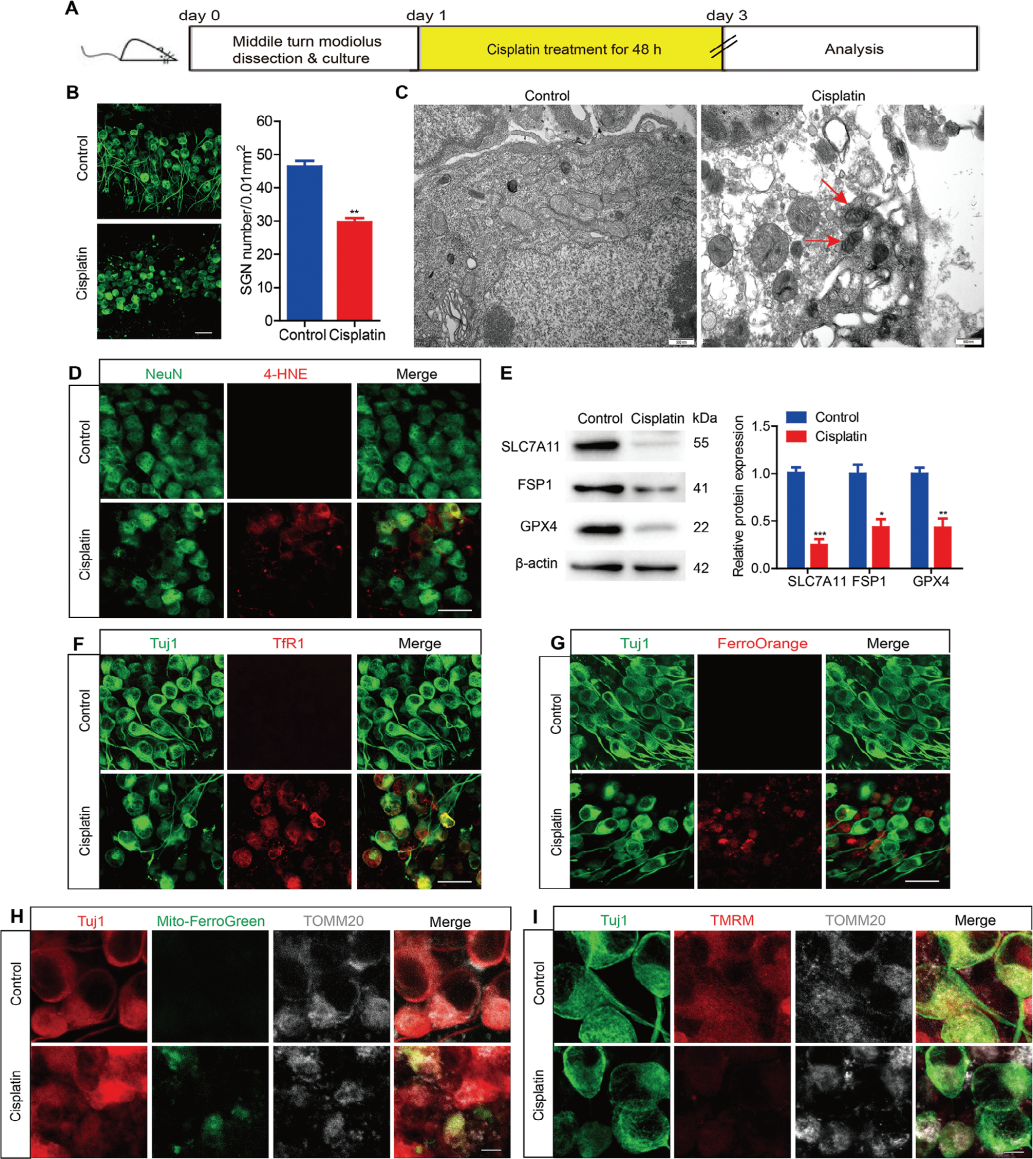
Cisplatin induces ferroptosis in cochlear SGNs in vitro. A) Cultured SGNs from P3 WT mice were exposed to 50 µM cisplatin for 48 h. B) Immunostaining and cell counting indicated that cisplatin treatment resulted in ≈64.02 ± 4.49% of SGNs remaining after treatment compared to the normal control group, n = 6 samples for each group. Scale bar = 25 µm. C) TEM showed that cisplatin led to mitochondrial shrinkage, a decrease or loss of mitochondrial cristae, and an increase in mitochondrial membrane density. The damaged mitochondria are indicated by the red arrows. n = 3 samples for each group. Scale bar = 500 nm. D) Immunostaining demonstrated that the fluorescence intensity of 4‐HNE was significantly higher in SGNs treated with cisplatin than those in the control group, n = 6 samples for each group. Scale bar = 25 µm. E) Western blot analysis showed that the protein levels of SLC7A11, FSP1 and GPX4 in SGNs were markedly decreased after cisplatin treatment, n = 3 samples. F–H) Immunofluorescence staining revealed stronger TfR1 F), FerroOrange G) and Mito‐FerroGreen H) fluorescences in the cisplatin‐treated group than those in the control group, n = 4 samples for each group. F, G: scale bar = 25 µm. H: scale bar = 5 µm. I) The TMRM fluorescence intensity was significantly decreased in cisplatin‐treated SGNs, n = 4 samples. Scale bar = 5 µm. The data are presented as the mean ± S.D. *p < 0.05, **p < 0.01, ***p < 0.001, two‐tailed, unpaired Student's t‐tests.

In vitro cisplatin treatment was carried out according to our previous report.^[^ ^13^, ^14^ ^]^ Specifically, cultured cochlear SGNs were exposed to 50 µM cisplatin for 48 h, resulting in ≈64.02 ± 4.49% of SGNs remaining after treatment compared to the normal control group (**Figure** **1**A,B). Representative images from TEM analysis (Figure 1C) revealed that cisplatin led to characteristic morphological changes related to ferroptosis in SGNs, including mitochondrial shrinkage, a decrease or loss of mitochondrial cristae, and an increase in mitochondrial membrane density. A key indicator of ferroptosis is intracellular lipid peroxidation. We measured the level of 4‐HNE, a widely recognized marker of lipid peroxidation. As shown in Figure 1D, the fluorescence intensity of 4‐HNE was significantly increased in SGNs following cisplatin injury compared to that in the control group. The expression levels of ferroptosis‐related proteins, including SLC7A11, FSP1, and GPX4, were markedly decreased after cisplatin treatment (Figure 1E). Considering that ferroptosis is an iron‐dependent form of programmed cell death, we evaluated the expression of transferrin receptor 1 (TfR1) and measured the accumulation of intracellular iron and mitochondrial iron in cisplatin‐treated SGNs using the probes FerroOrange and Mito‐FerroGreen, respectively. The immunofluorescence results (Figure 1F–H) demonstrated stronger fluorescence signals of TfR1, FerroOrange, and Mito‐FerroGreen in the cisplatin‐treated group than those in the control group, indicating excessive iron accumulation in SGNs due to cisplatin damage. Moreover, the mitochondrial membrane potential was assessed using the TMRM staining assay, and the results indicated a significant decrease in TMRM fluorescence intensity in cisplatin‐treated SGNs, signifying a decrease in the mitochondrial membrane potential (Figure 1I). Taken together, these findings indicate that cisplatin induces ferroptosis in cochlear SGNs in vitro.

**Figure 2:**
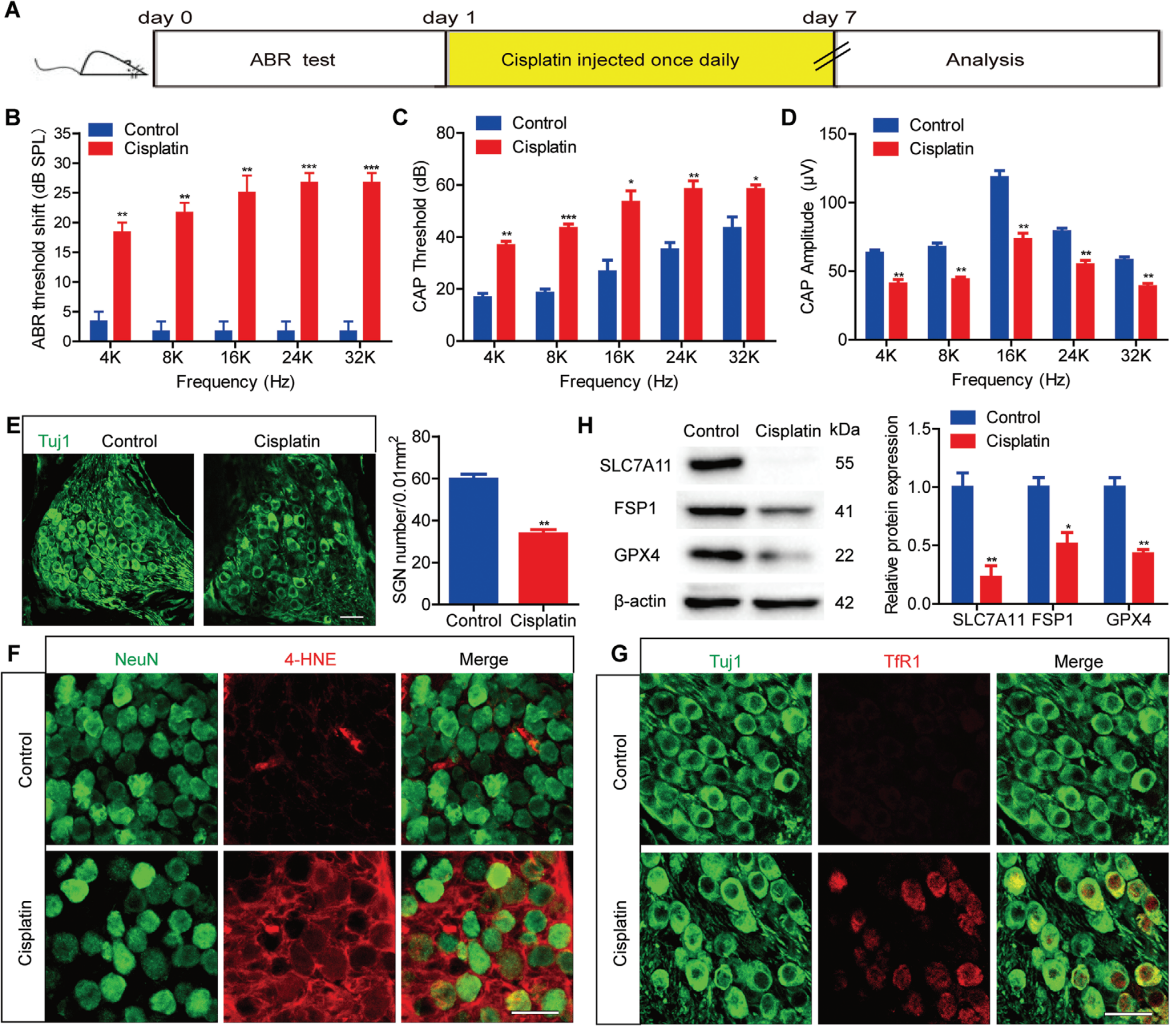
Cisplatin induces ferroptosis in cochlear SGNs in vivo. A) WT C57BL/6 mice were treated with 3 mg/kg cisplatin for 7 consecutive days by intraperitoneal (i.p.) injection beginning at P30. B,C) Cisplatin administration led to significant increases in the ABR threshold B) and CAP threshold C) at all tested frequencies, n = 6 mice. D) Cisplatin administration reduced in the CAP amplitude at 90 dB SPL across all tested frequencies, n = 6 mice. E) Immunostaining showed that cisplatin caused significant SGN loss in the cochlea, n = 6 samples. Scale bar = 25 µm. F,G) Cisplatin administration significantly upregulated 4‐HNE F) and TfR1 G), n = 4 samples for each group. Scale bar = 25 µm. H) Western blot analysis revealed that cisplatin treatment downregulated the expressions of SLC7A11, FSP1 and GPX4 in cochlear SGNs in vivo, n = 3 samples. The data are presented as the mean ± S.D. *p < 0.05, **p < 0.01, ***p < 0.001, two‐tailed, unpaired Student's t‐tests or two‐way ANOVA with a post‐hoc Student Newman‐Keuls test.

We then investigated whether cisplatin induced ferroptosis in cochlear SGNs in vivo. Wild‐type (WT) C57BL/6 mice were administered 3 mg kg^−1^ of cisplatin for seven consecutive days. This treatment led to significant increases in the auditory brainstem response (ABR) thresholds and central auditory processing (CAP) thresholds across all tested frequencies from 4 to 32 kHz (**Figure** **2**A–C), along with a noticeable reduction in the CAP amplitude at 90 dB SPL across all frequencies (Figure 2D), confirming hearing loss in the mice. Neuronal class III β‐tubulin (Tuj1) staining and cell counting indicated that cisplatin caused significant SGN loss in the mouse cochlea (Figure 2E). In line with the in vitro findings, cisplatin treatment resulted in significant upregulation of 4‐HNE and TfR1, and downregulation of SLC7A11, FSP1, and GPX4 in cochlear SGNs in vivo (Figure 2F–H). These results suggest that cisplatin‐induced damage also triggers ferroptosis in cochlear SGNs in vivo.

### 2.2 Inhibition of Ferroptosis Prevents Cisplatin‐Induced SGN Loss and Hearing Loss In Vitro and In Vivo

**Figure 3:**
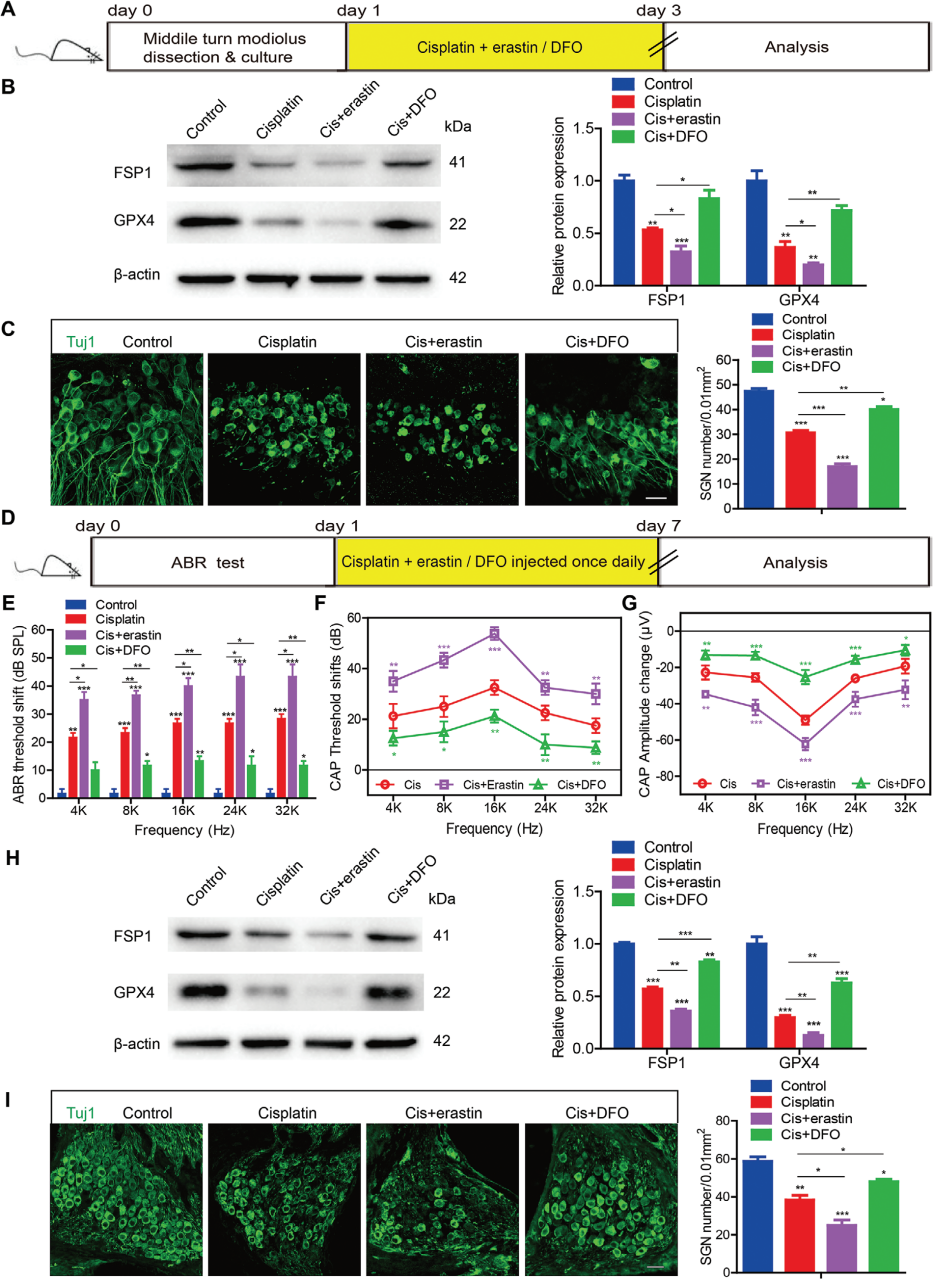
Inhibition of ferroptosis prevents cisplatin‐induced SGN loss and hearing loss. A) Cultured SGNs from P3 WT mice were treated with 50 µM cisplatin for 48 h in the presence or absence of the ferroptosis activator erastin (25 µM) or the ferroptosis inhibitor DFO (80 µM). B) Western blot analysis showed that cisplatin treatment caused a reduction of FSP1 and GPX4 in SGNs compared to the control group. Pretreatment with erastin in the Cis + erastin group significantly exacerbated these reductions, while pretreatment with DFO in the Cis + DFO group migrated them compared to the cisplatin‐only group. n = 3 samples. C) Increased SGN loss was observed following erastin pretreatment and cisplatin injury compared to the cisplatin treatment alone group, but this loss was attenuated by pretreatment with DFO. n = 6 samples for each group. Scale bar = 25 µm. D) C57BL/6 mice were i.p. injected with 3 mg/kg cisplatin daily for 7 days with or without erastin or DFO co‐treatment starting at P30. E,F) The ABR threshold shift E) and CAP threshold shift F) were much larger in the Cis + erastin group, whereas they were smaller in the Cis + DFO group than in the cisplatin only group. n = 6 mice. G) DFO alleviated the reduction in the CAP amplitude in cisplatin‐treated mice, whereas erastin administration had the opposite effect. n = 6 mice. H) Cisplatin administration in mice induced downregulations of FSP1 and GPX4 in SGNs compared with the control mice, and the expressions of FSP1 and GPX4 were further decreased in the Cis + erastin group, while they were increased in the Cis + DFO group, compared to the cisplatin only group. n = 3 samples. I) Cell counting revealed fewer surviving SGNs in the Cis + erastin group but significantly more SGNs in the Cis + DFO group than in the cisplatin alone group. n = 6 samples for each group. Scale bar = 25 µm. The data are presented as the mean ± S.D. *p < 0.05, **p < 0.01, ***p < 0.001, one‐way ANOVA with Tukey's post‐hoc test or two‐way ANOVA with a post‐hoc Student Newman‐Keuls test. DFO, deferoxamine.

To explore the role of ferroptosis in cisplatin‐induced damage to SGNs and hearing loss, a ferroptosis activator, erastin, and a ferroptosis inhibitor, the iron chelator deferoxamine (DFO), were co‐administered with cisplatin to cultured SGNs and mice, respectively. In vitro experiments revealed that pretreatment with erastin significantly increased the TfR1 expression and FerroOrange signals, as well as exacerbated the reduction of TMRM in SGNs treated with cisplatin, while DFO exerted opposite effects in SGNs after exposure to cisplatin (Figure S1A–D, Supporting Information). Correspondingly, the protein levels of FSP1 and GPX4 were decreased in the Cis + erastin group but were increased in the Cis + DFO group compared to those in the cisplatin only group (**Figure** **3**A,B). Immunostaining and cell counting indicated less SGNs were survived in the Cis + erastin group compared to cisplatin treatment alone group, while the SGNs loss was mitigated by DFO pretreatment (Figure 3C). These results demonstrate that erastin effectively activates, while DFO inhibits ferroptosis in cisplatin‐treated SGNs, thereby exacerbating or preventing cisplatin‐induced SGN loss in vitro.

In vivo experiments were conducted in which the mice were administered cisplatin alone or in combination with erastin or DFO once daily (Figure 3D). Hearing function was assessed after drug treatments, and both ABR and CAP threshold shifts were significantly higher in the Cis + erastin group and lower in the Cis + DFO group than in the cisplatin only group (Figure 3E,F). Additionally, co‐treatment with erastin markedly exacerbated, while cotreatment with DFO mitigated, the reduction in CAP amplitudes in cisplatin‐treated mice (Figure 3G). The protein levels of FSP1 and GPX4 were decreased in the Cis + erastin group but increased in the Cis + DFO group, relative to those in the cisplatin only group (Figure 3H). Cell counting results indicated fewer surviving SGNs in the Cis + erastin group, but significantly more SGNs in the Cis + DFO group than in the cisplatin alone group (Figure 3I). Overall, these findings substantiate that ferroptosis inhibition can prevent cisplatin‐induced SGN loss and hearing loss in mice.

### 2.3 Ferritinophagy is Activated in Cochlear SGNs after Cisplatin Treatment

**Figure 4:**
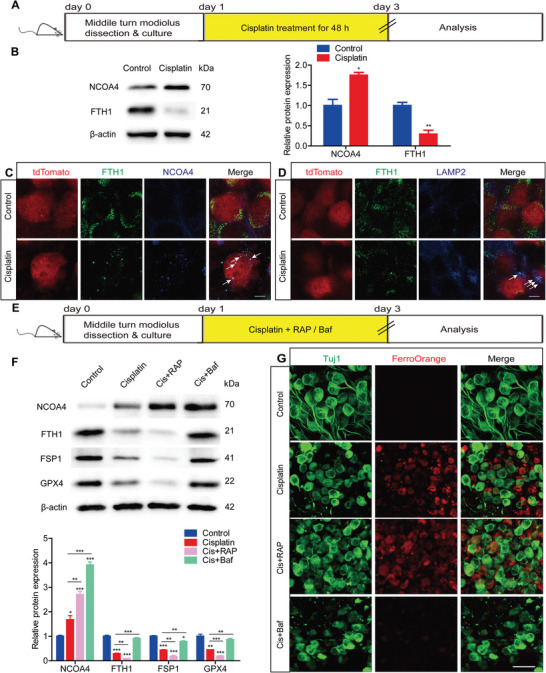
Ferritinophagy is activated in cochlear SGNs after cisplatin treatment. A) Cultured SGNs from P3 WT mice or Bhlhb5‐cre/Rosa26‐tdTomato mice were exposed to 50 µM cisplatin for 48 h. B) Western blot analysis showed an increase in NCOA4 expression and a decrease in FTH1 expression in cultured SGNs after cisplatin treatment. n = 3 samples. C) Immunofluorescence showed colocalization of NCOA4 and FTH1 in cisplatin‐treated SGNs. n = 4 samples for each group. Scale bar = 5 µm. D) The colocalization of FTH1 and LAMP2 was increased in SGNs after cisplatin injury. n = 4 samples for each group. Scale bar = 5 µm. E) Cultured SGNs from P3 WT mice were treated with 50 µM cisplatin for 48 h alone or with the autophagy activator RAP (0.1 µM) or the autophagy inhibitor Baf (100 nM). F,G) RAP significantly upregulated NCOA4 protein expression and downregulated FTH1 expression, resulting in increased accumulation of intracellular iron and reductions in FSP1 and GPX4 protein levels in SGNs treated with cisplatin. Baf co‐treatment had the opposite effect. n = 3 samples for each group. Scale bar = 25 µm. The data are presented as the mean ± S.D. *p < 0.05, **p < 0.01, ***p < 0.001, two‐tailed, unpaired Student's t‐tests or one‐way ANOVA with Tukey's post‐hoc test. RAP, rapamycin; Baf, bafilomycin A1.

Iron released from ferritin via ferritinophagy, which is mediated by NCOA4 through lysosomal degradation, is a primary source of intracellular iron. Given the significant increase in intracellular iron in SGNs following cisplatin injury, we evaluated the activation of ferritinophagy in cochlear SGNs after cisplatin exposure. Initially, we observed an increase in NCOA4 expression and a decrease in ferritin heavy chain 1 (FTH1) expression in cultured SGNs after cisplatin treatment (**Figure** **4**A,B). Subsequently, we assessed the combination of NCOA4 with FTH1 and the transport of this complex to the lysosome in cisplatin‐treated SGNs using immunostaining. Due to the unavailability of suitable antibodies for triple staining of SGNs, FTH1 with NCOA4 or lysosomes, we utilized tdTomato‐labeled SGNs derived from Bhlhb5‐cre/Rosa26‐tdTomato mice, as previously published by our group.^[^ ^39^ ^]^ Immunofluorescence analysis revealed that significant colocalization of NCOA4 and FTH1 was observed in cisplatin‐treated SGNs (Figure 4C), indicating an increase in NCOA4‐FTH1 complex fusion in SGNs following cisplatin exposure. Furthermore, the co‐labeling of FTH1 and LAMP2, a known lysosomal marker, was also increased in SGNs after cisplatin‐induced injury (Figure 4D), suggesting that cisplatin induced the association of ferritin with lysosomes in SGNs. Together, our results indicate that ferritinophagy is activated in cisplatin‐treated SGNs.

Ferritinophagy, a form of selective autophagy, prompted further investigation into the effect of autophagy on ferroptosis in cochlear SGNs induced by cisplatin. Experiments utilizing the autophagy activator rapamycin (RAP) and the autophagy inhibitor bafilomycin A1 (Baf) in cisplatin‐damaged SGNs revealed that RAP significantly upregulated NCOA4 protein expression and downregulated FTH1 expression. This resulted in an increased accumulation of intracellular iron and reductions in FSP1 and GPX4 protein levels in SGNs treated with cisplatin (Figure 4E–G). Baf, functioning as an inhibitor of autophagosome and lysosome fusion, blocks the degradation of autophagosomes. Our findings indicated that Baf treatment markedly increased the protein expression levels of NCOA4 and FTH1, significantly mitigated cisplatin‐induced iron accumulation, and increased the protein expression of FSP1 and GPX4 (Figure 4E–G). These results confirm that the activation of autophagy can promote cisplatin‐induced ferritinophagy and ferroptosis, while the inhibition of autophagy markedly alleviates these processes in SGNs.

### 2.4 NCOA4 Knockdown Rescues Cisplatin‐Induced SGN Loss and Ferroptosis in Cultured SGNs

**Figure 5:**
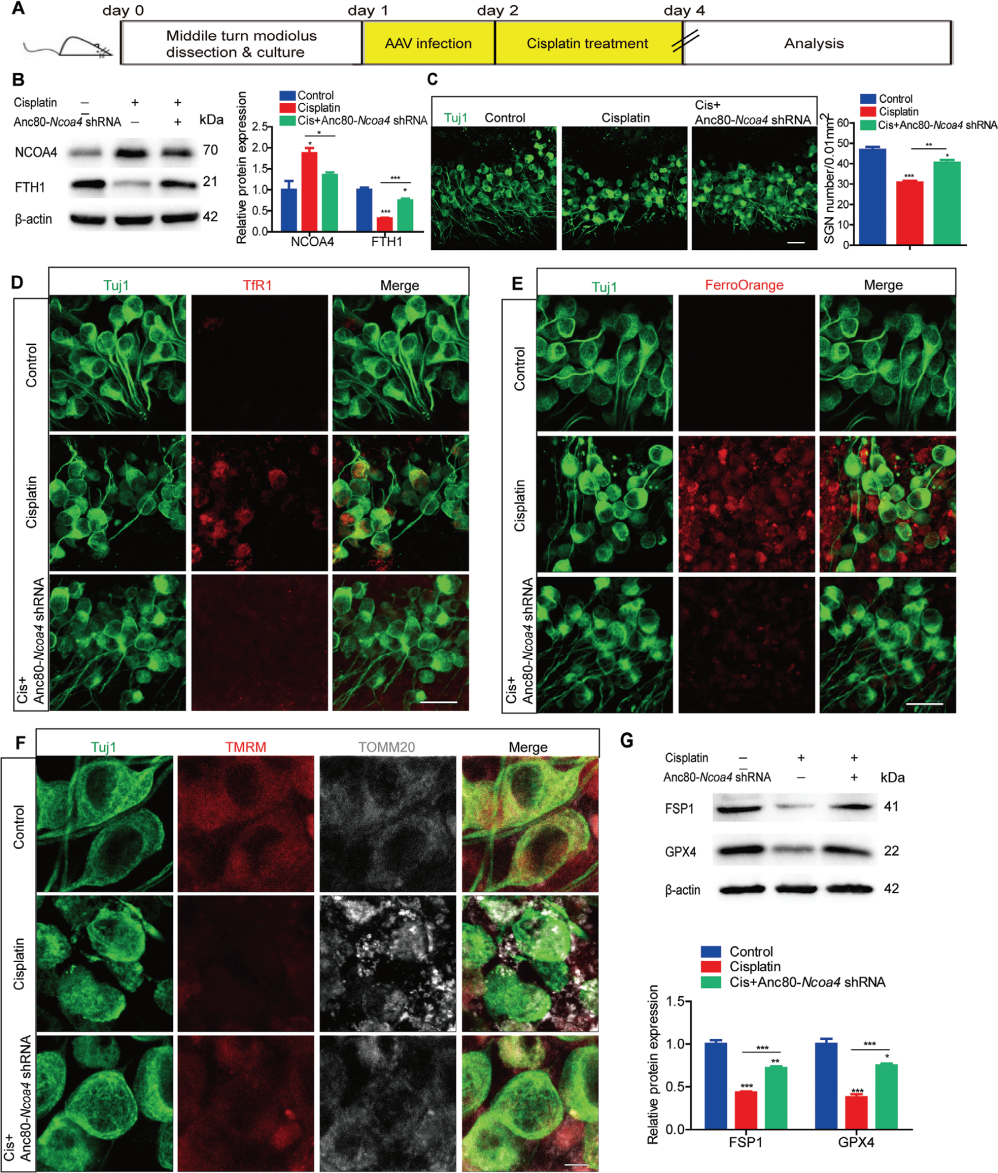
NCOA4 knockdown rescues cisplatin‐induced SGN loss and ferroptosis in cultured SGNs. A) Cultured SGNs from P3 WT mice were incubated with 4 × 10^10^ GC mL^−1^ Anc80‐*Ncoa4* shRNA for 24 h. Then, the medium was replaced with normal medium, and the SGNs were further incubated with 50 µM cisplatin for 48 h. B) Western blot analysis revealed that the increase in NCOA4 expression and decrease in FTH1 expression in SGNs treated with cisplatin were significantly alleviated by pretreatment with Anc80‐*Ncoa4* shRNA. n = 3 samples. C) More surviving SGNs were found in the Cis + Anc80‐*Ncoa4*‐shRNA group than in the cisplatin only group. n = 6 samples for each group. Scale bar = 25 µm. D,E) NCOA4 knockdown significantly reduced the expression of TfR1 D) and the FerroOrange intensity E) in cisplatin‐treated SGNs. n = 4 samples for each group. Scale bar = 25 µm. F,G) NCOA4 knockdown increased the TMRM signal intensity F) and the protein levels of FSP1 and GPX4 G) in cisplatin‐treated SGNs compared with SGNs in the cisplatin only group. n = 3 samples for each group. Scale bar = 5 µm. The data are presented as the mean ± S.D. *p < 0.05, **p < 0.01, ***p < 0.001, one‐way ANOVA with Tukey's post‐hoc test.

To investigate the role of NCOA4‐mediated ferritinophagy in cisplatin‐induced ferroptosis in SGNs, we conducted NCOA4 inactivation experiments in cultured SGNs using Anc80‐*Ncoa4* shRNA (**Figure** **5**A). Two shRNAs targeting *Ncoa4* were designed, and shRNA‐*Ncoa4*‐2 was selected for subsequent experiments based on its relatively superior knockdown efficiency (Figure S2C, Supporting Information). Our findings exhibited that ≈60% of SGNs were successfully transfected with Anc80‐*Ncoa4* shRNA, significantly reduced NCOA4 expression in cultured SGNs (Figure S2A–C, Supporting Information). The observed increase in NCOA4 expression and decrease in FTH1 expression in SGNs following cisplatin exposure were significantly mitigated by pretreatment with Anc80‐*Ncoa4* shRNA (Figure 5B), resulting in a higher survival rate of SGNs in the Cis + Anc80‐*Ncoa4* shRNA group than in the cisplatin only group (Figure 5C). The knockdown of NCOA4 significantly decreased the expression of TfR1 and the intensity of FerroOrange, while it increased the expression of TMRM and the protein levels of FSP1 and GPX4 in cisplatin‐treated SGNs relative to those in the cisplatin only group (Figure 5D–G). In conclusion, NCOA4 knockdown rescues cisplatin‐induced ferroptosis and cell loss in cultured SGNs.

### 2.5 NCOA4 Knockdown Alleviates Cisplatin‐Induced Hearing Loss, SGN Loss and Ferroptosis in Mice

**Figure 6:**
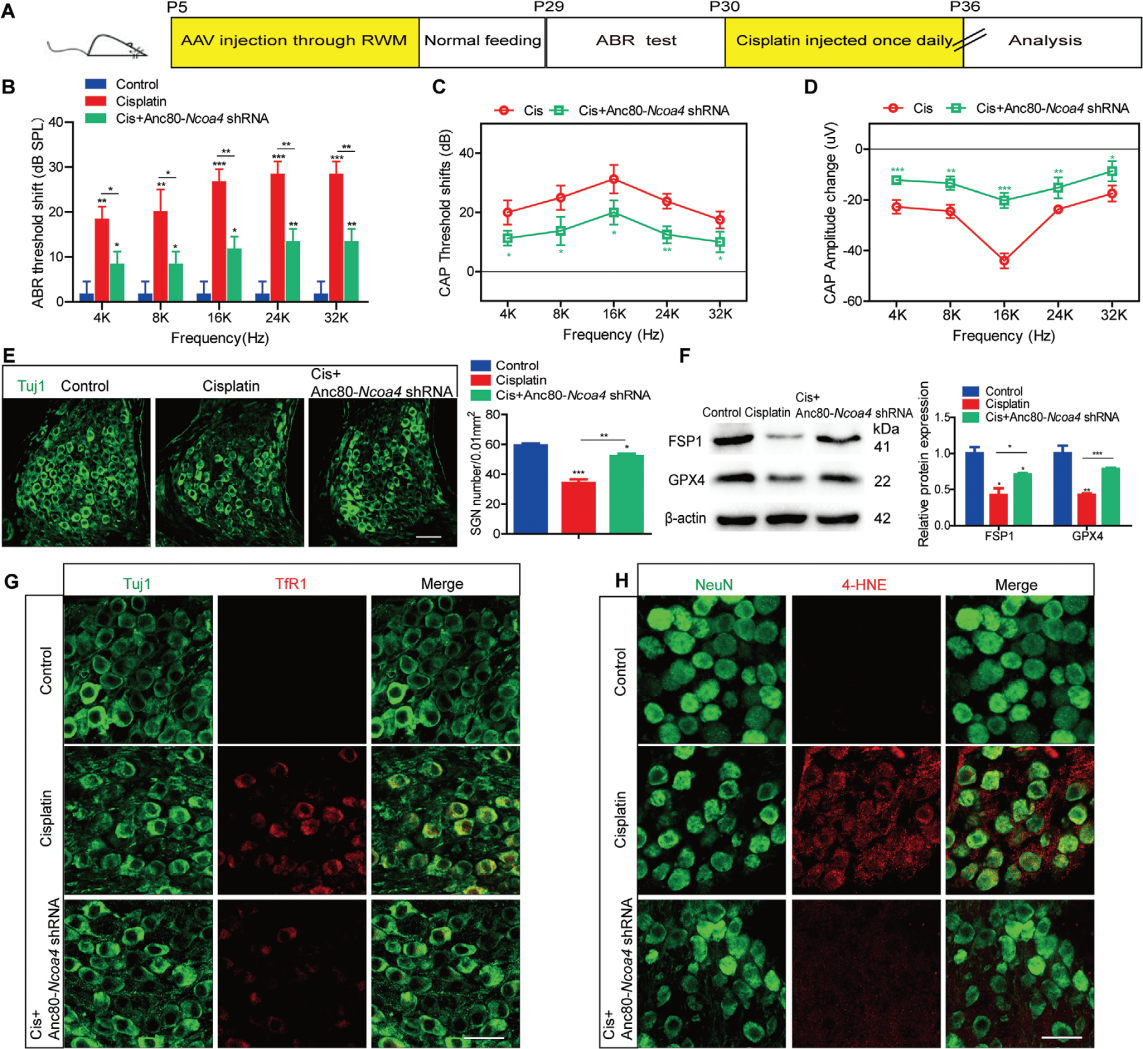
NCOA4 knockdown alleviates cisplatin‐induced hearing loss, SGN loss and ferroptosis in mice. A) WT mice were injected with 1 × 10^10^ GCs of Anc80‐*Ncoa4* shRNA via the RWM at P5 and then administered 3 mg kg^−1^ cisplatin by i.p. injection for 7 consecutive days beginning at P30. B–D) The shifts in the ABR threshold B) and CAP threshold C) in the Cis + Anc80‐*Ncoa4* shRNA group were significantly inhibited compared to those in the cisplatin only group, whereas the change in the CAP amplitude D) was increased in the Cis + Anc80‐*Ncoa4* shRNA group. n = 6 mice. E) Cell counting revealed more surviving SGNs in the Cis+Anc80‐*Ncoa4* shRNA group than in the cisplatin only group. n = 6 samples for each group. Scale bar = 25 µm. F) The protein levels of FSP1 and GPX4 in mice cotreated with cisplatin and Anc80‐*Ncoa4* shRNA were increased compared to those in mice treated with cisplatin alone. n = 3 samples. G,H) The increase in TfR1 G) and 4‐HNE H) fluorescence intensity in cisplatin‐treated SGNs was markedly inhibited by pretreatment with Anc80‐*Ncoa4* shRNA. n = 4 samples for each group. Scale bar = 25 µm. The data are presented as the mean ± S.D. *p < 0.05, **p < 0.01, ***p < 0.001, one‐way ANOVA with Tukey's post‐hoc test or two‐way ANOVA with a post‐hoc Student Newman‐Keuls test.

We further evaluated the effect of NCOA4 knockdown on hearing function and SGN survival in mice in vivo. After Anc80‐*Ncoa4* shRNA was injected into the cochlea via the round window membrane (RWM) at P5, ≈60% of the SGNs in the apical, middle, and basal turns exhibited mNeonGreen fluorescence, respectively. The expression of NCOA4 was significantly reduced in the inner ears of P30 mice (Figure S2D–G, Supporting Information). After coadministration with cisplatin, the shifts in the ABR threshold and CAP threshold were significantly inhibited, whereas the change in the CAP amplitude was increased in the Cis + Anc80‐*Ncoa4* shRNA group compared to those in the cisplatin only group (**Figure** **6**A–D). Cell counting revealed that there were significantly more surviving SGNs in the Cis + Anc80‐*Ncoa4* shRNA group than in the cisplatin only group (Figure 6E). Consistent with the results shown in vitro, NCOA4 knockdown also upregulated the protein levels of FSP1 and GPX4 in mice cotreated with cisplatin and Anc80‐*Ncoa4* shRNA compared to those in mice treated with cisplatin alone (Figure 6F). Moreover, the increase in the TfR1 and 4‐HNE fluorescence intensity in SGNs induced by cisplatin was markedly inhibited by pretreatment with Anc80‐*Ncoa4* shRNA (Figure 6G,H). Therefore, these results indicate that knockdown of NCOA4 can alleviate cisplatin‐induced hearing loss, SGN loss, and ferroptosis in mice.

### 2.6 FOXO1 Binds to the Promoter of NCOA4, Suppresses NCOA4 Expression and Inhibits Ferritinophagy and Ferroptosis Induced by Cisplatin in SGNs

**Figure 7:**
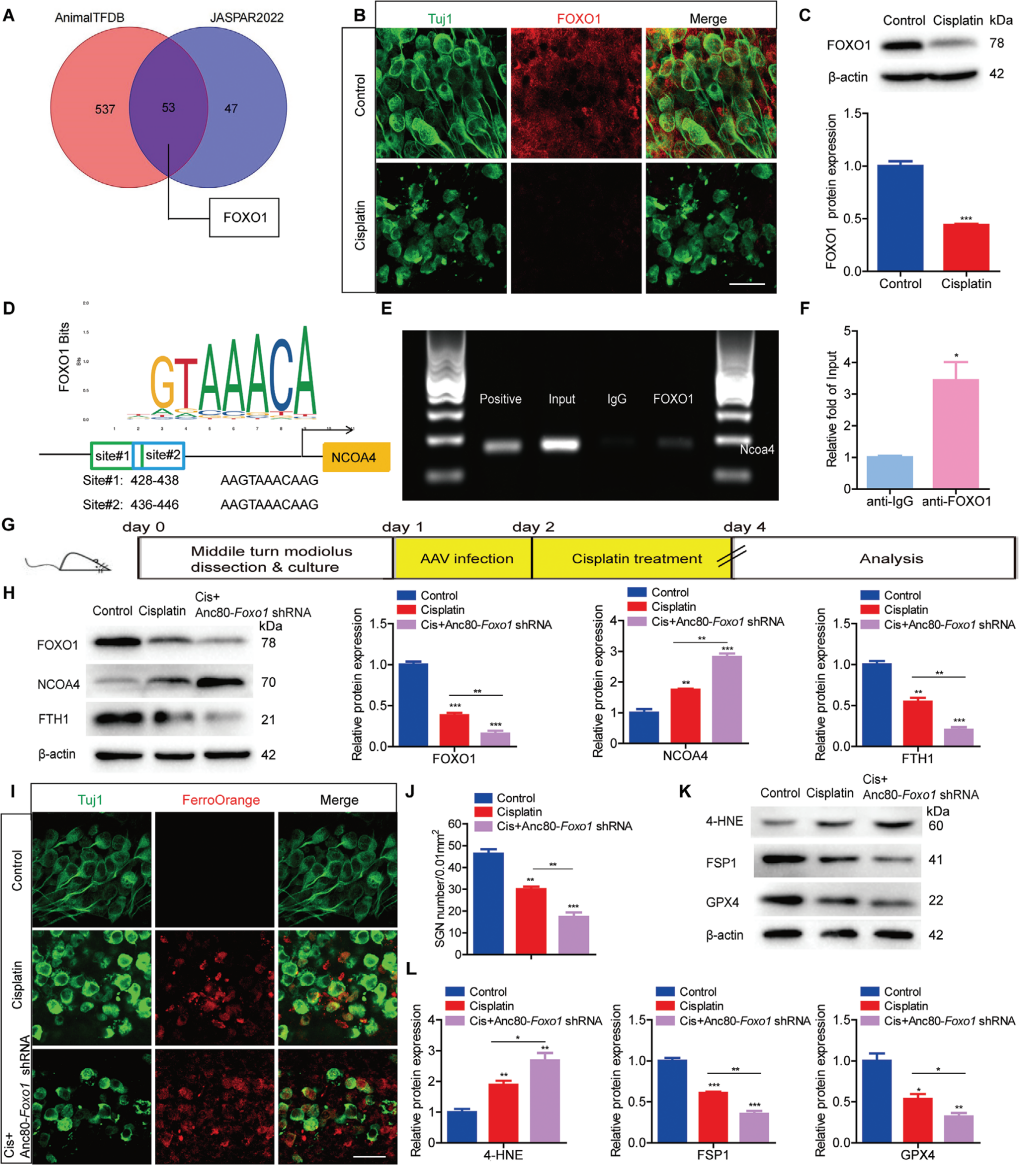
FOXO1 binds to the promoter of NCOA4, suppresses NCOA4 expression, and inhibits ferritinophagy and ferroptosis in cisplatin‐treated SGNs. A) Two TF prediction websites (JASPAR and Animal TFDB) were used to conduct bioinformatics analysis and predict the potential TFs that bind to the promoter sequence of *Ncoa4*. B,C) Immunofluorescence staining B) and Western blot C) revealed that the expression level of FOXO1 was significantly decreased in cisplatin‐treated cultured SGNs. n = 3 samples for each group. Scale bar = 25 µm. D) As predicted by the JASPAR database, there were two potential FOXO1 binding sites in the NCOA4 promoter. E,F) ChIP‒PCR showed that FOXO1 bound directly to the NCOA4 promoter. n = 3 samples. G) Cultured SGNs from P3 WT mice were incubated with 4 × 10^10^ GC mL^−1^ Anc80‐*Foxo1* shRNA for 24 h. Then, the medium was replaced with normal medium, and the SGNs were further incubated with 50 µM cisplatin for 48 h. H) Western blot analysis revealed that the expression of FOXO1 was further reduced, the expression of NCOA4 was increased, and the expression of FTH1 was decreased in the Cis + Anc80‐*Foxo1* shRNA group SGNs compared to SGNs in the cisplatin only group. n = 3 samples. I–L) FOXO1 deficiency increased intracellular iron accumulation I), aggravated SGN loss J), increased 4‐HNE protein level and further reduced FSP1 and GPX4 protein levels K,L) in the Cis + Anc80‐*Foxo1* shRNA group than in the cisplatin only group. n = 3 samples for each group. Scale bar = 25 µm. The data are presented as the mean ± S.D. *p < 0.05, **p < 0.01, ***p < 0.001, two‐tailed, unpaired Student's t‐tests or one‐way ANOVA with Tukey's post‐hoc test.

As cisplatin induced a significant upregulation of NCOA4, thereby activating ferritinophagy in SGNs, we further aimed to identify possible upstream regulators of NCOA4 in SGNs. We utilized two TF prediction websites, JASPAR and Animal TFDB, for bioinformatics analysis to predict potential TFs that could bind to the promoter sequence of *Ncoa4*. Overall, 53 overlapping candidate TFs were identified by both algorithms (**Figure** **7**A). Among these TFs, FOXO1, known for its role in modulating oxidative stress and maintaining homeostasis in various diseases, has been reported to be associated with ferroptosis.^[^ ^33^, ^36^, ^38^ ^]^ Our findings were supported by immunofluorescence staining and western blot analysis and demonstrated a significant decrease in FOXO1 expression in cisplatin‐treated SGNs, suggesting FOXO1's involvement in cisplatin‐induced damage to SGNs (Figure 7B, C). To elucidate the regulatory relationship between FOXO1 and NCOA4 in SGNs, we first investigated whether FOXO1 could directly bind to the NCOA4 promoter. According to predictions from the JASPAR database, two potential FOXO1 binding sites on the NCOA4 promoter were identified (Figure 7D). A chromatin immunoprecipitation (ChIP) assay, using chromatin derived from SGNs and subsequent PCR amplification, was performed to confirm these sites. Given that these two sites partially overlapped, complicating the design of distinct primers for each site, we designed *Ncoa4* primers that could amplify the sequence encompassing both FOXO1 sites. PCR revealed a distinct band amplified by the *Ncoa4* primers in samples that precipitated with an anti‐FOXO1 antibody. Quantitative analysis indicated a significant increase in NCOA4 enrichment in the anti‐FOXO1 group compared to the negative control anti‐IgG antibody group, demonstrating FOXO1's direct binding to the NCOA4 promoter (Figure 7E,F). Additionally, sequencing data from the ChIP‒PCR products provided further evidence of FOXO1 occupancy on the NCOA4 promoter (Figure S3A,B, Supporting Information).

Subsequently, we examined the role of FOXO1 in regulating NCOA4‐mediated ferritinophagy and ferroptosis in SGNs. The expression of FOXO1 was effectively decreased in cultured SGNs following Anc80‐*Foxo1* shRNA transfection (Figure S3C–E, Supporting Information). The co‐administration of cisplatin and knockdown of FOXO1 via Anc80‐*Foxo1* shRNA further reduced FOXO1 expression, increased NCOA4 expression, and decreased FTH1 expression in SGNs compared to those in the cisplatin only group (Figure 7G,H), indicating that NCOA4 expression was negatively regulated by FOXO1 and that FOXO1 inhibition enhanced ferritinophagy in SGNs treated with cisplatin. Furthermore, FOXO1 knockdown increased intracellular iron accumulation, aggravated SGN loss, increased 4‐HNE protein level and further reduced the protein levels of FSP1 and GPX4 in the Cis + Anc80‐*Foxo1* shRNA group compared to those in the cisplatin only group (Figure 7I–L). Together, these findings confirm that FOXO1 can directly target NCOA4 to suppress its expression and that inhibiting FOXO1 exacerbates NCOA4‐mediated ferritinophagy, ferroptosis, and lipid peroxidation in SGNs treated with cisplatin.

### 2.7 Disruption of the FOXO1‐NCOA4 Axis Prevents Cisplatin‐Induced SGN Ferroptosis and Hearing Loss in Mice

**Figure 8:**
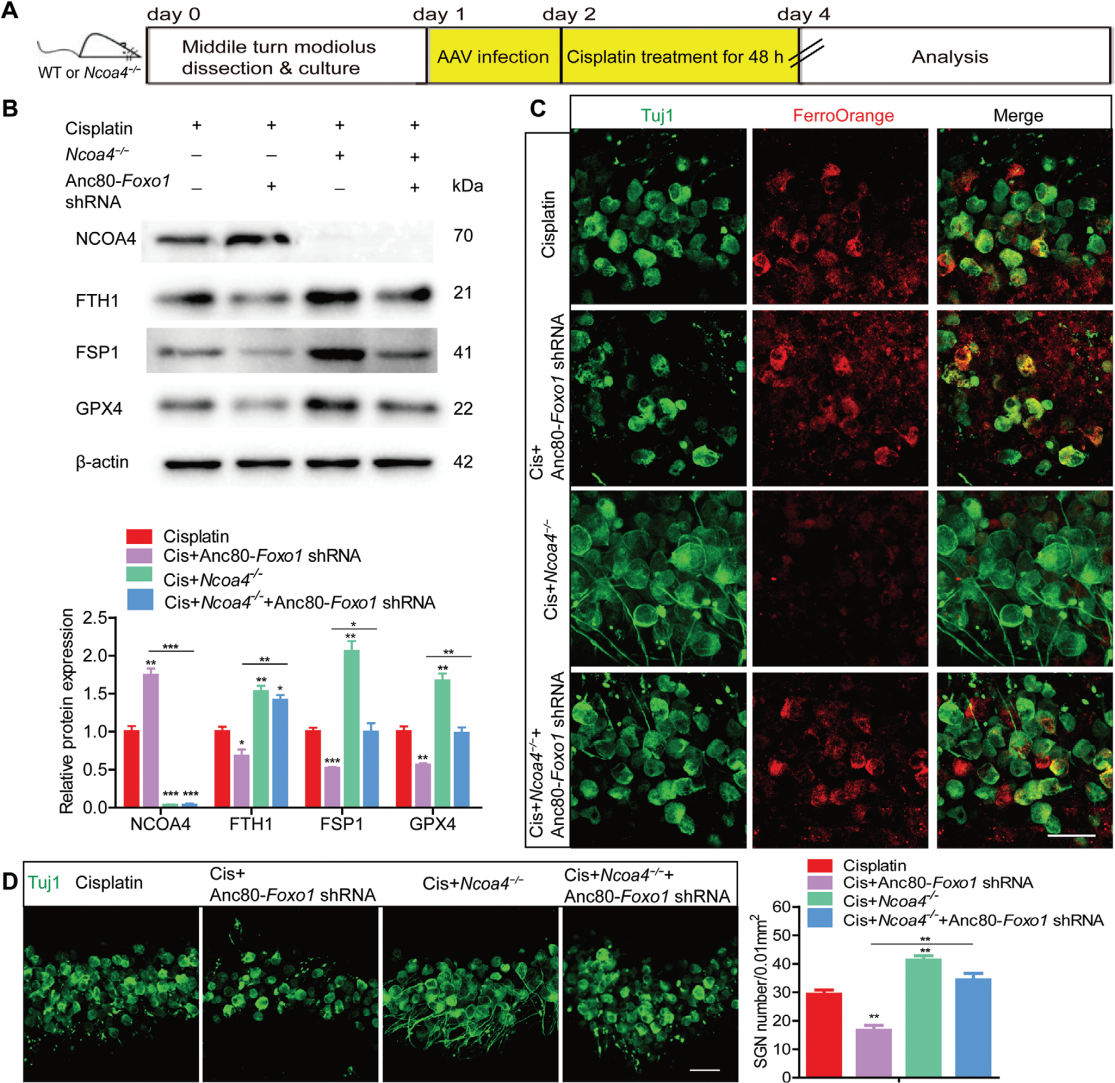
Disruption of the FOXO1‐NCOA4 axis rescues cisplatin‐induced SGN loss and ferroptosis in cultured SGNs. A) Cultured SGNs from P3 WT mice or *Ncoa4* ^−/−^ mice were incubated with 4 × 10^10^ GC mL^−1^ Anc80‐*Foxo1* shRNA for 24 h, after which the medium was replaced with normal medium. The SGNs were then further incubated with 50 µM cisplatin for 48 h. B,C) Genetic knockout of NCOA4 significantly increased FTH1 expression, decreased intracellular iron accumulation, and upregulated FSP1 and GPX4 protein expression in *Ncoa4* ^−/−^ mouse SGNs after cisplatin treatment. NCOA4 deficiency inhibited the accumulation of intracellular iron and the reduction in FSP1 and GPX4 levels in the Cis + *Ncoa4* ^−/−^ + Anc80‐*Foxo1* shRNA group compared with the Cis + Anc80‐*Foxo1* shRNA group. n = 3 samples for each group. Scale bar = 25 µm. D) The number of surviving SGNs was significantly greater in the Cis + *Ncoa4* ^−/−^ group than in the cisplatin only group. NCOA4 deficiency increased the number of surviving SGNs in the Cis + *Ncoa4* ^−/−^ + Anc80‐*Foxo1* shRNA group compared with the Cis + Anc80‐*Foxo1* shRNA group. n = 4 samples for each group. Scale bar = 25 µm. The data are presented as the mean ± S.D. *p < 0.05, **p < 0.01, ***p < 0.001, one‐way ANOVA with Tukey's post‐hoc test.

To clarify the role of the FOXO1‐NCOA4 axis in cisplatin‐induced SGN damage and hearing loss, we further conducted rescue experiments with *Ncoa4* knockout (*Ncoa4* ^−/−^) mice under the knockdown of FOXO1. First, we examined the expression of NCOA4 in *Ncoa4* ^−/−^ mice and found it to be almost undetectable in cochlear SGNs, with no change in FOXO1 expression (Figure S4A,B, Supporting Information) or hearing loss detected in *Ncoa4* ^−/−^ mice. This suggested that the absence of NCOA4 did not affect auditory function or FOXO1 expression. Similar to the results obtained for cultured SGNs in the Cis + Anc80‐*Ncoa4* shRNA group (Figure 5), genetic knockout of NCOA4 significantly increased FTH1 expression, decreased intracellular iron accumulation, and upregulated the protein expression of FSP1 and GPX4 in *Ncoa4* ^−/−^ mouse SGNs after cisplatin treatment (**Figure** **8**A–C). Notably, the decreases in FTH1, FSP1 and GPX4 levels caused by the inhibition of FOXO1 via Anc80‐*Foxo1* shRNA were reversed in the Cis + *Ncoa4* ^−/−^ + Anc80‐*Foxo1* shRNA group compared to those in the Cis + Anc80‐*Foxo1* shRNA group (Figure 8B,C). Furthermore, NCOA4 deficiency invalidated the enrichment of intracellular iron and increased the number of surviving SGNs in the Cis + *Ncoa4* ^−/−^ + Anc80‐*Foxo1* shRNA group compared with the Cis + Anc80‐*Foxo1* shRNA group (Figure 8C,D).

**Figure 9:**
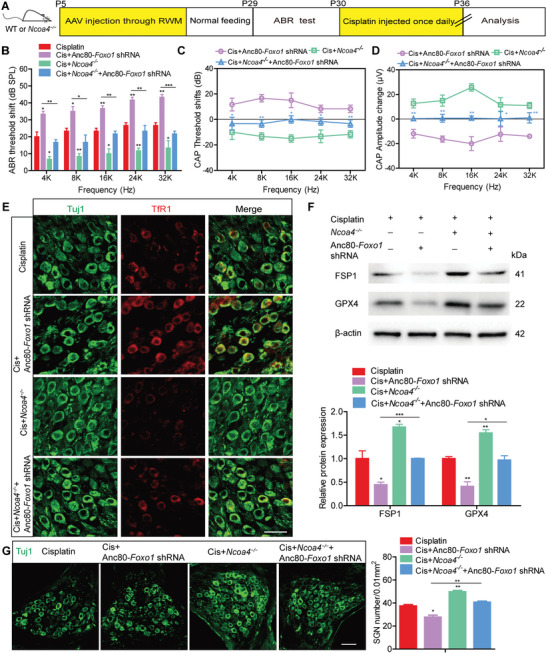
Disruption of the FOXO1‐NCOA4 axis rescues cisplatin‐induced SGN ferroptosis and hearing loss in mice. A) WT mice or *Ncoa4* ^−/−^ mice were injected with 1 × 10^10^ GCs of Anc80‐*Foxo1* shRNA via the RWM at P5, followed by administration of 3 mg/kg cisplatin via i.p. injection for 7 consecutive days beginning at P30. B–D) Increases in the ABR thresholds B) and CAP thresholds C), along with a decrease in CAP amplitude D), were observed in the Cis + Anc80‐*Foxo1* shRNA group compared with the cisplatin‐only group. Hearing function was improved in the Cis + *Ncoa4* ^−/−^ + Anc80‐*Foxo1* shRNA mice relative to the Cis + Anc80‐*Foxo1* shRNA mice. n = 6 mice. E,F) FOXO1 knockdown significantly increased TfR1 expression E), and decreased the protein levels of FSP1 and GPX4 F) in mice treated with cisplatin. These protein changes were reversed in the Cis + *Ncoa4* ^−/−^ + Anc80‐*Foxo1* shRNA group compared to the Cis + Anc80‐*Foxo1* shRNA group. n = 3 samples for each group. Scale bar = 25 µm. G) FOXO1 knockdown reduced the number of surviving SGNs in WT mice treated with cisplatin, while significantly more SGNs were observed in the Cis + *Ncoa4* ^−/−^ + Anc80‐*Foxo1* shRNA group than in the Cis + Anc80‐*Foxo1* shRNA group. n = 4 samples for each group. Scale bar = 25 µm. Data are presented as the mean ± S.D. *p < 0.05, **p < 0.01, ***p < 0.001, one‐way ANOVA with Tukey's post‐hoc test or two‐way ANOVA with a post‐hoc Student Newman‐Keuls test.

Finally, we investigated the role of the FOXO1‐NCOA4 axis in cochlear SGNs in vivo. Anc80‐*Foxo1* shRNA was injected into mouse cochleae via the RWM at P5, which significantly reduced FOXO1 expression in the inner ears of P30 mice (Figure S4C–F, Supporting Information). Co‐administration of cisplatin with the Anc80‐*Foxo1* shRNA to WT mice exacerbated hearing loss, as evidenced by significant increases in the ABR and CAP thresholds and a decrease in the CAP amplitude in the Cis + Anc80‐*Foxo1* shRNA group compared to the cisplatin only group. However, hearing loss was mitigated in NCOA4‐deficient mice cotreated with cisplatin and Anc80‐*Foxo1* shRNA, with improved hearing function in the Cis + *Ncoa4* ^−/−^ + Anc80‐*Foxo1* shRNA group compared to the Cis + Anc80‐*Foxo1* shRNA group (**Figure** **9**A–D). Consistent with in vitro results, FOXO1 knockdown significantly increased TfR1 expression and decreased the protein levels of FSP1 and GPX4 in mice co‐treated with cisplatin and Anc80‐*Foxo1* shRNA, compared to those treated with cisplatin alone. Conversely, the increase in TfR1 expression and the decreases in FSP1 and GPX4 levels observed in the Cis + Anc80‐*Foxo1* shRNA group were reversed in NCOA4‐deficient mice in the Cis + *Ncoa4* ^−/−^ + Anc80‐*Foxo1* shRNA group (Figure 9E,F). This indicates that FOXO1 inhibition exacerbated ferroptosis in SGNs of mice exposed to cisplatin while this effect could be blocked by NCOA4 deficiency. Fewer surviving SGNs were observed in WT mice co‐treated with cisplatin and Anc80‐*Foxo1* shRNA compared to the cisplatin only group (Figure 9G). In contrast, significantly more SGNs were present in the Cis + *Ncoa4* ^−/−^ + Anc80‐*Foxo1* shRNA group than in the Cis + Anc80‐*Foxo1* shRNA group (Figure 9G). Together, these results suggest that NCOA4 is a target gene of FOXO1, and disruption of the FOXO1‐NCOA4 axis ameliorated cisplatin‐induced SGN ferroptosis, thereby rescuing the SGNs loss and hearing loss in mice exposed to cisplatin.

## 3 Discussion

Cisplatin exposure can cause SGN injury, leading to SNHL and hindering the effectiveness of cochlear implants. To date, research has shown that the ototoxicity of cisplatin induces apoptosis, necrosis, and autophagy in inner ear cells, ^[^ ^7^, ^8^, ^12^, ^14^ ^]^ ultimately resulting in cell death. However, inhibiting apoptosis and necrosis can only partially restore auditory cell damage caused by cisplatin and the subsequent hearing impairment. Therefore, auditory cell death due to cisplatin and the underlying mechanisms require further investigation. Here, our study revealed that cisplatin treatment also initiated classic ferroptotic markers in SGNs. These markers include alterations in mitochondrial ultrastructure, increased levels of intracellular and mitochondrial iron, heightened lipid peroxidation, and decreased expression of ferroptosis signature proteins. Thus, our research provides the inaugural evidence of cisplatin‐induced ferroptosis in cochlear SGNs, both in vitro and in vivo.

Cisplatin, a platinum‐based chemotherapeutic agent, exhibits a high affinity for thiol‐rich biomolecules. Within the cytoplasm, it binds to GSH, a prevalent non‐protein thiol, forming a Pt‐GS complex and resulting in GSH depletion.^[^ ^40^, ^41^ ^]^ This process mirrors the action of the ferroptosis inducer erastin, where GSH depletion and GPX suppression underlie cisplatin cytotoxicity.^[^ ^41^, ^42^ ^]^ Previous studies have reported cisplatin‐induced ferroptosis in various cell types, including cancer cells, renal tubular epithelial cells, and cochlear HCs,^[^ ^43^, ^44^, ^45^ ^]^ which is aligned with our findings. Furthermore, our study demonstrated that inhibiting ferroptosis significantly reduced SGNs damage and mitigated hearing loss in cisplatin‐treated mice, while activation of ferroptosis led to worse outcomes. Inhibition of ferroptosis using iron chelators and lipophilic antioxidants has emerged as a protective strategy and a promising therapeutic approach for various diseases.^[^ ^46^, ^47^, ^48^ ^]^ In auditory research, the application of Fer‐1 or DFO has been shown to protect cochlear HCs from cisplatin‐induced damage in vitro ^[^ ^22^, ^23^, ^32^ ^]^ and in animal models including mice,^[^ ^32^ ^]^ rats ^[^ ^49^ ^]^ and zebrafish.^[^ ^23^ ^]^ These results underscore the therapeutic potential of ferroptosis inhibition in preserving inner ear cells and restoring hearing following cisplatin‐induced ototoxicity.

Ferritinophagy, a critical process that regulates cellular iron homeostasis and ROS production,^[^ ^50^ ^]^ acts as a precursor to ferroptosis and is implicated in the development of various conditions, including cancer,^[^ ^51^ ^]^ neurodegenerative diseases,^[^ ^52^ ^]^ anemia,^[^ ^53^ ^]^ cardiovascular disease,^[^ ^54^ ^]^ and others.^[^ ^55^ ^]^ Its activation has been observed in HEI‐OC1 cells following cisplatin treatment ^[^ ^32^ ^]^ or NPC1 deficiency,^[^ ^56^ ^]^ and it has been reported that nuciferine can protect HCs from ferroptosis via inhibiting NCOA4‐mediated ferritinophagy.^[^ ^57^ ^]^ However, the specific roles and regulatory mechanisms of ferritinophagy in auditory neurons are not yet understood. Here, we found that NCOA4‐mediated ferritinophagy was initiated in cochlear SGNs treated with cisplatin. Silencing NCOA4 halted FTH1 degradation, decreased intracellular iron levels, mitigated ferroptosis, and ultimately preserved hearing in mice. Pharmacological inhibition of autophagy with Baf significantly reduced ferritin degradation and ferroptosis in SGNs exposed to cisplatin, confirming that cisplatin‐induced ferroptosis in SGNs was autophagy dependent. Interestingly, this conclusion contrasts with our previous findings, where cisplatin‐induced autophagy activation in SGNs was shown to play a protective role against apoptosis.^[^ ^14^ ^]^ This discrepancy highlights a complex relationship where enhancing autophagy can paradoxically increase ferritinophagy and exacerbate ferroptosis in SGNs. Therefore, targeted NCOA4 knockout, combined with autophagy activation, might offer a more effective approach for hearing restoration.

Given the stimulatory effect of NCOA4‐mediated ferritinophagy on ferroptosis, targeting upstream regulators of this cascade, including dysregulated iron levels and ROS production, to modulate the NCOA4 signaling pathway is a promising strategy for treating ferroptosis‐associated pathologies. Research has demonstrated that NCOA4 regulation is influenced by factors such as iron levels,^[^ ^58^ ^]^ autophagy,^[^ ^28^ ^]^ lysosomes,^[^ ^59^ ^]^ and hypoxia.^[^ ^50^ ^]^ Upstream TFs such as hypoxia inducible factor‐1α (HIF‐1α),^[^ ^60^ ^]^ HIF‐2α,^[^ ^61^ ^]^ STAT3,^[^ ^62^ ^]^ and JUN^[^ ^63^ ^]^ have also been verified to directly target NCOA4 expression. These interactions influence iron mobilization, contribute to conditions such as osteoarthritis, and can exacerbate cardiac injury in specific models. Our study observed that FOXO1, a widely present TF, was downregulated in SGNs following cisplatin exposure. FOXO1 binds to the NCOA4 promoter, directly regulating its expression. Silencing FOXO1 aggravated NCOA4‐mediated ferritinophagy, increased SGN sensitivity to cisplatin‐induced ferroptosis, and thereby exacerbated hearing loss in mice. More importantly, disrupting the FOXO1‐NCOA4 axis in NCOA4 knockout mice halted this deterioration. Recent studies have highlighted the role of FOXO1 in ferroptosis, demonstrating its inhibitory effect on GPX4, which not only prevents prostate cancer metastasis but also induces ferroptosis in cervical cancer cells.^[^ ^36^, ^37^ ^]^ Additionally, FOXO1‐induced upregulation of FTH1 has been shown to inhibit ferroptosis in cardiomyocytes.^[^ ^38^ ^]^ Our findings align with these reports, indicating FOXO1's involvement in inhibiting ferroptosis and revealing a novel mechanism by which FOXO1 counteracts NCOA4‐mediated ferritinophagy and subsequent ferroptosis. However, evidence from another study suggested that FOXO1 promotes ferroptosis in pancreatic β cells in type 2 diabetes mellitus, where FOXO1 knockdown reduced ROS, iron, and ACSL4 levels while increasing GPX4 levels.^[^ ^64^ ^]^ This suggests the complex and context‐dependent relationship between FOXO1 and ferroptosis, likely influenced by the specific downstream targets regulated by FOXO1. Therefore, to further understand the protective mechanism against cisplatin‐induced ferroptosis in SGNs, the regulation of other downstream targets by FOXO1, in addition to NCOA4, needs to be verified in future studies.

**Figure 10:**
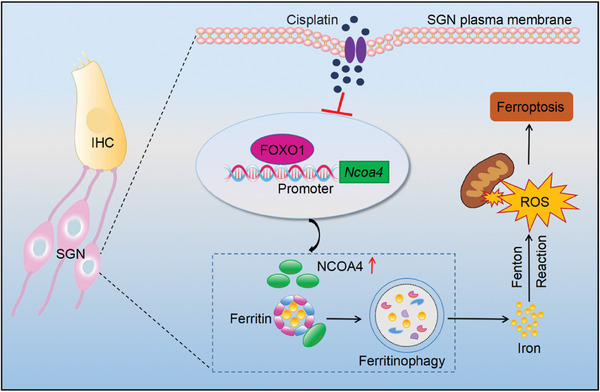
A graphic illustration for the role of FOXO1‐NCOA4 axis in cisplatin‐induced SGNs ferroptosis and hearing loss. Cisplatin exposure induces ROS production and triggers ferroptosis in SGNs. The TF FOXO1, which is downregulated in response to cisplatin exposure, binds to the NCOA4 promoter enhancing its expression. Elevated NCOA4 levels increase ferritinophagy, thereby releasing free iron and driving Fenton reaction, ultimately leading to ferroptosis in SGNs. This diagram highlights the potential intervention points where inhibiting FOXO1 or NCOA4 could mitigate ferroptosis and thus reducing hearing loss associated with cisplatin treatment. IHC, inner hair cell; SGN, spiral ganglion neuron; FOXO1, forkhead box transcription factor O1; NCOA4, nuclear receptor coactivator 4; ROS, reactive oxygen species.

In summary, our study found that exposure to cisplatin triggers ferroptosis in SGNs in vitro and in vivo. Inhibiting ferroptosis protected against cisplatin‐induced damage to SGNs and subsequent hearing loss. Cisplatin activated NCOA4‐mediated ferritinophagy in SGNs, and FOXO1 acts as a direct upstream regulator of NCOA4, disrupting the FOXO1‐NCOA4 axis prevented ferroptosis in SGNs and hearing loss induced by cisplatin (**Figure** **10**). The protective role of FOXO1 against ferroptosis and hearing loss is, at least in part, dependent on its ability to modulate NCOA4‐mediated ferritinophagy. From a clinical perspective, our findings offer significant insights into potential treatments aimed at ameliorating cisplatin‐induced damage to SGNs via inhibiting ferroptosis. In particular, pharmacological candidates that target NCOA4 or FOXO1, as a regulator of ferritinophagy and iron homeostasis, appear promising for mitigating cisplatin ototoxicity and SNHL. These candidates can be screened and tested through in vitro and animal experiments. However, due to genomic and physiological differences between mice and humans, the results from mouse model studies may not fully translate to human conditions. Developing potential clinical treatments for SNHL will require a considerable amount of time and numerous studies.

## 4 Experimental Section

### Animals and Genotyping

WT C57BL/6J mice were obtained from the Animal Center of Shandong University (Jinan, China). *Ncoa4* ^−/−^ mice on the C57BL/6 background were purchased from Shanghai Model Organisms Center, Inc. (NM‐KO‐2105583, Shanghai, China). Bhlhb5‐cre mice were generously provided by Dr. Lin Gan (University of Rochester, New York), and Rosa26‐tdTomato reporter mice were purchased from the Jackson Laboratory (stock no. 007914). Genotyping was performed using PCR on genomic DNA extracted from mouse tail tips, which were incubated in 70 µL of 50 mM NaOH at 98 °C for 1 h, followed by neutralization in 7 µL of 1 M HCl. The specific genotyping primers used were detailed in **Table** **1**. This animal studies adhered to protocols sanctioned by the Animal Care Committee of Shandong University, China (No. ECAESDUSM 20123011), and aligned with the National Institute of Health's Guide for the Care and Use of Laboratory Animals. The animals were maintained in a climate‐controlled environment at 23 ± 2 °C on a 12/12 h light/dark cycle.

**Table 1:** PCR primer sequences used in the experiments.

Gene	Forward sequence	Reverse sequence
Ncoa4‐WT	TCTGTGTGCTTGGAGAGGTAA	CCACCATCCATGAGGTCAGA
Ncoa4‐Mut	GGTTTCTCTTCCCGTAGCCC	ACTCGGTTGCCTGTCCAAAT
Bhlhb5‐cre	GGGATTGGACTCAGAGGCGGTAGC	GCCCAAATG TTGCTGGATAT
tdTomato‐WT	AAGGGAGCTGCAGTGGAGTA	CCGAAAATCTGTGGGAAGTC
tdTomato‐Mut	GGCATTAAAGCAGCGTATCC	CTGTTCCTGTACGGCATGG

### Organotypic Culture of Mouse Cochlear SGNs and Drug Treatments In Vitro

The mice were dissected on postnatal day 3 (P3), at which the cochlea modiolus was easier to be dissected out and maintain in good shape, as well as before it was calcified and ossified, as described in the previously published reports.^[^ ^13^, ^14^ ^]^ For consistency across experiments, only the middle turns of the mouse cochlea were harvested and cultured. Isolated middle turn explants, which contained SGNs, were incubated overnight at 37 °C in a 5% CO_2_ atmosphere in Dulbecco's modified Eagle medium/F12 (DMEM/F12; Gibco, 11330032) supplemented with 10% fetal bovine serum (FBS; Gibco, 10091148) and 50 mg ml^−1^ ampicillin (Sigma, A5354).

The next day, the culture media of the SGN explants was replaced with media supplemented with cisplatin (50 µM, Sigma‒Aldrich, P4394) with or without the following agents for 48 h: erastin (25 µM; Selleck, S7242), deferoxamine (DFO, 80 µM; Selleck, S5742), bafilomycin A1 (Baf, 100 nM; Abcam, ab120497), and rapamycin (RAP, 0.1 µM; Abcam, ab120224). After the culture period, the samples were collected for subsequent assays.

### TEM Analysis

After cisplatin treatment, the cultured SGN explants were first pre‐fixed with 3% glutaraldehyde, followed by fixation with 1% osmium tetroxide. The samples were then dehydrated in a graded acetone series and embedded in Epon 812. For optical localization, semithin sections were stained with methylene blue. Ultrathin sections were prepared using a diamond knife and stained with uranyl acetate and lead citrate. The sections were examined with a JEM‐1400‐FLASH transmission electron microscope at Chengdu Lilai Biotechnology Co., Ltd. (Chengdu, China).

### Iron Ion Detection

To detect intracellular and mitochondrial iron levels, the probes FerroOrange (Dojindo, F374) and Mito‐FerroGreen (Dojindo, M489) were utilized, respectively. Following various treatments, the cultured SGN explants were incubated with 1 µmol L^−1^ FerroOrange or 5 µmol L^−1^ Mito‐FerroGreen for 30 min at 37 °C in a 5% CO_2_ atmosphere. A confocal laser scanning microscope (Leica SP8; Leica, Germany) was used to capture images.

### Mitochondrial Membrane Potential Measurement

To assess the mitochondrial membrane potential in SGN explants, we used TMRM (Invitrogen, I34361) according to the manufacturer's instructions. The explants were treated for the specified time, stained with 200 nmol L^−1^ TMRM in the dark for 30 min at 37 °C, and examined with a confocal laser scanning microscope (Leica SP8; Leica, Germany).

### In Vivo Drug Administration

The mice used for in vivo experiments were at P30, at which the hearing function of mice is fully developed and mature for hearing analysis. The cisplatin dose and treatment procedure were chose based on our previous studies which can effectively induce hearing loss and SGN damage with the least lethality in mice.^[^ ^13^, ^14^ ^]^ It was sourced healthy WT C57BL/6J mice of P30 with normal hearing and randomly assigned them to various groups for i.p. injections. The control group received 0.9% saline daily for 7 days, starting at P30. The cisplatin group was administered 3 mg kg^−1^ cisplatin daily for the same duration. In the cisplatin + erastin group, the mice received i.p. injections of erastin (20 mg kg^−1^; Selleck, S7242) 2 h before each cisplatin injection, starting from P30. Similarly, the cisplatin + DFO group was given i.p. injections of DFO (100 mg kg^−1^; Selleck, S5742) 2 h prior to each cisplatin injection for 7 consecutive days beginning at P30.

### In Vitro Virus Administration

NCOA4 or FOXO1 expression was knocked down using AAV vectors. It was engineered recombinant viruses using the AAV vector Anc80L65, which carried shRNA sequences targeting mouse *Ncoa4* or *Foxo1*: Anc80L65‐mNeonGreen‐*Ncoa4*‐shRNA‐HA (Anc80‐*Ncoa4* shRNA) and Anc80L65‐mNeonGreen‐*Foxo1*‐shRNA‐HA (Anc80‐*Foxo1* shRNA). The efficiency of AAV‐mediated transfection and knockdown of NCOA4 or FOXO1 was first assessed. Cultured cochlear SGNs from P3 WT mice were treated with 4 × 10^10^ GC mL^−1^ Anc80‐*Ncoa4* shRNA or Anc80‐*Foxo1* shRNA for 24 h. Afterward, the medium was replaced with fresh normal medium, and the SGNs were incubated for an additional 48 h before proceeding to immunostaining or Western blot analysis. Additionally, to evaluate the effect of NCOA4 or FOXO1 on ferritinophagy and ferroptosis, cochlear SGNs from P3 WT mice or *Ncoa4* ^−/−^ mice were cultured with 4 × 10^10^ GC mL^−1^ Anc80‐*Ncoa4* shRNA or Anc80‐*Foxo1* shRNA for 24 h. The medium was subsequently exchanged for normal medium, and the SGNs were incubated with 50 µM cisplatin for 48 h before further analysis.

### In Vivo Virus Administration

Viral injections through the round window membrane RWM were performed as outlined in prior studies.^[^ ^65^ ^]^ Briefly, P5 WT mice were anesthetized on ice. Surgery was performed on the left ear, and the right ear served as a control. An incision behind the ear allowed for the dissection of tissue to access the otic bulla and expose the RWM. Virus injections were then administered through the RWM using a glass micropipette (25 µm) over 120 s, with the volume of injected virus being ≈0.33 µL per cochlea. This process was precisely controlled by the UMP3 UltraMicro Pump (World Precision Instrument). Then, a veterinary tissue adhesive (Millpledge Ltd., UK) was used to seal the incision. The mice were then warmed in an incubator at 30 °C.

For drug administration, mice in the cisplatin + Anc80‐*Ncoa4* shRNA group or the cisplatin + Anc80‐*Foxo1* shRNA group received injections of 1 × 10^10^ GCs of Anc80‐*Ncoa4* shRNA or Anc80‐*Foxo1* shRNA into the left scala tympani through the RWM of the left ear. Subsequently, they were i.p. administered 3 mg kg^−1^ cisplatin daily for seven consecutive days, starting at P30.

### Auditory Function Tests

The auditory function of the mice was assessed using ABR and CAP tests. The mice were anesthetized with an intramuscular injection of ketamine (50 mg kg^−1^) and xylazine (6 mg kg^−1^). For ABR testing, electrodes were positioned as follows: a recording electrode was inserted at the apex of the skull, a reference electrode was placed subcutaneously behind the test ear, and a grounding electrode was inserted subcutaneously behind the opposite ear. The TDT System III (Tucker‐Davis Technologies, USA) was employed for stimulus production and response recording. ABR was elicited using tone bursts at frequencies of 4, 8, 16, 24, and 32 kHz, with 1024 stimulus repetitions and a scanning time of 10 ms. The initial stimulation intensity was set at 90 dB SPL and decreased by 5 dB steps until the response was no longer detectable. The lowest intensity eliciting a response was recorded as the hearing threshold.

For CAP testing, a silver ball electrode was placed on the RWM. TDT System III produced tone bursts at frequencies of 4, 8, 16, 24, and 32 kHz, each with a 10 ms duration and 0.5 ms rise/fall time. A preamplifier (RA16PA, TDT) amplified the cochlear signal 500 times, averaging over 512 repetitions. The CAP was filtered and displayed. The stimulus intensity was reduced in 5 dB steps from 90 to 0 dB SPL. The intensity that triggered a 3 µV CAP amplitude was defined as the CAP threshold. The CAP amplitude, calculated as the voltage difference between the first negative (N1) and subsequent positive peak (P1), was plotted against stimulus intensity to produce an input/output (I/O) function at each tested frequency.

### Cryosectioning

Cochleae were removed from adult mice and fixed with 4% paraformaldehyde (PFA) at 4 °C overnight. The samples were then decalcified in EDTA decalcification solution and incubated in 10%, 20%, and 30% sucrose. Subsequently, the tissues were embedded in O.C.T., quickly frozen on dry ice, and stored at −80 °C. A cryostat (Leica CM 1850; Leica, Germany) was used to cut the frozen tissues into 7 µm sections.

### Immunostaining

Following incubation or cryosection, SGN explants or tissue sections were fixed with 4% PFA, permeabilized with 1% Triton X‐100 for 15 min, and blocked with PBT‐1 solution (0.1% Triton X‐100, 8% donkey serum, 1% bovine serum albumin, and 0.02% sodium azide in PBS) at room temperature for 1 h. The samples were then incubated overnight at 4 °C with the following primary antibodies diluted in PBT‐1 solution: anti‐Tuj 1 (1:1000, Abcam, ab78078), anti‐NeuN (1:500, Cell Signaling Technology, 12943), anti‐4‐HNE (1:500, Abcam, ab48506), anti‐TfR1 (1:400, ABclonal, A5865), anti‐TOMM20 (1:400, Proteintech, 11802‐1‐AP), anti‐NCOA4 (1:400, Abnova, H00008031‐M04 and 1:1000, Affinity, DF4255), anti‐FTH1 (1:400, Abcam, ab183781), anti‐LAMP2 (1:400, Abcam, ab25631), and anti‐FOXO1 (1:400, CST, 2880S). The following day, the samples were incubated with FITC‐conjugated or TRITC‐conjugated secondary antibodies (1:1000, Invitrogen) and DAPI (Sigma‒Aldrich, D9542) in the dark at room temperature for 1 h. Finally, the samples were mounted and examined using a laser scanning confocal microscope (Leica SP8; Leica, Germany).

### Western Blotting

Total proteins were extracted from cultured SGNs using RIPA buffer (Protein Biotechnology, China) for the in vitro samples. For the in vivo experiments involving adult cochlear SGNs, the basilar membrane and other tissues were meticulously removed to isolate only the modiolus of the cochlea. These tissues were then homogenized using a low‐temperature tissue grinding instrument (Jingxin JXFSTPRP‐CL, Shanghai, China) in RIPA buffer (Protein Biotechnology, China), after which the supernatant was collected.

Each protein sample was separated by SDS‒PAGE and subsequently transferred onto a PVDF membrane. The primary antibodies applied included anti‐SLC7A11 (1:1000 dilution, Affinity, DF12509), anti‐FSP1 (1:1000 dilution, Proteintech, 20886‐1‐AP), anti‐GPX4 (1:1000 dilution, Abcam, ab125066), anti‐NCOA4 (1:1000 dilution, Affinity, DF4255), anti‐FTH1 (1:1000 dilution, Abcam, ab183781), anti‐FOXO1 (1:1000 dilution, CST, 2880S), and anti‐β‐actin (1:2000 dilution, ZSGB‐BIO, TA‐09). The bands were visualized using an enhanced chemiluminescence (ECL) Western blotting detection kit (EpiZyme, China) and analyzed with ImageJ software.

### ChIP Assays

The enrichment of FOXO1 in the promoter region of NCOA4 was examined using ChIP assay kits (CST, 9005S) following the manufacturer's instructions. Briefly, SGNs were fixed with formaldehyde to establish cross‐links between proteins and DNA. Sonication was performed to shear the DNA to an optimal size. The chromatin‐protein complexes were then incubated with the positive control Histone H3, the negative control normal rabbit IgG, or FOXO1 antibodies overnight at 4 °C with rotation. Subsequently, the samples were incubated with ChIP‐Grade Protein G Magnetic Beads for 2 h at 4 °C with rotation. Chromatin was eluted from the antibody/Protein G Magnetic complex, after which the DNA was reverse cross‐linked from the protein/DNA complexes. Finally, the DNA was purified using spin columns and stored for further standard PCR analysis.

### Standard PCR

The master reaction mixtures were prepared, and each PCR product was analyzed by 2% agarose gel electrophoresis using a 100 bp DNA marker. In addition, the PCR products of FOXO1‐enriched DNA underwent sequencing to verify the binding between FOXO1 and NCOA4.

### SGN Counting

The Tuj1 antibody was utilized to immunolabel SGNs, and images of the cultured middle‐turn cochlear explants were captured using a Leica confocal microscope (Leica SP8; Leica, Germany). The total number of SGNs in which the nucleus comprised at least 40% of the soma area was counted and calculated using ImageJ software. For the in vivo experiments, SGN quantification was performed on middle cochlear sections from P30 mice. The number of SGNs per section was divided by the area of the Rosenthal canal, and the total number of SGNs was calculated using ImageJ software. The density of the SGNs was determined per unit area (0.01 mm^2^).

### Statistical Analysis

All the experiments were conducted at least three times, and n represents the number of independent samples or mice from each subgroup. All data were analyzed using GraphPad Prism 8.0 software and the data are presented as the mean ± standard deviation (SD). Two‐tailed, unpaired Student's t‐tests were used to determine statistical significance when comparing two groups. One‐way ANOVA followed by Tukey's multiple comparisons test were used for comparing multiple groups. Two‐way ANOVA followed by a post‐hoc Student Newman‐Keuls test was used for functional hearing assessments. A value of *P* < 0.05 was considered statistical significance.

## Conflict of Interest

The authors declare no conflict of interest.

## Author Contributions

X.W., L.X., and Y.M. contributed equally to this work. H.W., W.L., and R.C. designed the project. X.W., L.X., Y.M., F.C., J.Z., M.W., W.A., Y.H., B.C. performed the experiments and acquired the data. X.W., and W.L. analyzed the results and wrote the manuscript.

## Supporting information

Supporting Information

## Data Availability

The data that support the findings of this study are available from the corresponding author upon reasonable request.
