# Supplementary material for: FOXO1‐NCOA4 Axis Contributes to Cisplatin‐Induced Cochlea Spiral Ganglion Neuron Ferroptosis via Ferritinophagy
Source: Adv Sci (Weinh). 2024 Aug 29;11(40):2402671. doi: 10.1002/advs.202402671 (PMC11515924; doi:10.1002/advs.202402671)
Supplement: Supplementary file 1 — Supporting Information [file ADVS-11-2402671-s001.docx]

Supporting Information 1

**FOXO1-NCOA4 Axis Contributes to Cisplatin-Induced Cochlea Spiral Ganglion Neuron Ferroptosis via Ferritinophagy**

*Xue Wang^#^, Lei Xu^#^, Yu Meng^#^, Fang Chen, Jinzhu Zhuang, Man Wang, Weibin An, Yuechen Han, Bo Chu, Renjie Chai^*^, Wenwen Liu^*^, Haibo Wang^*^*


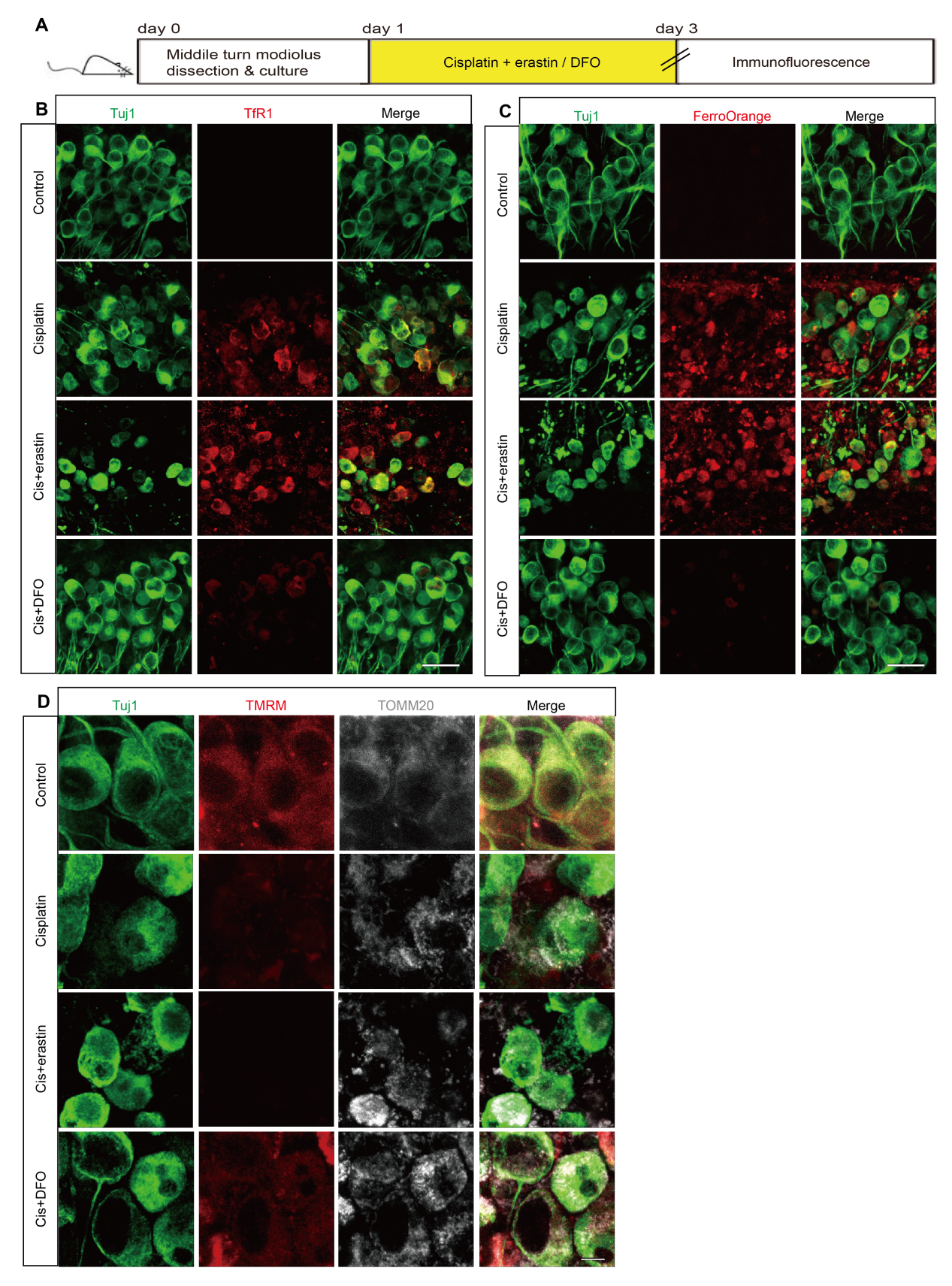


**Figure S1.** Effects of the ferroptosis activator erastin and the ferroptosis inhibitor DFO. A) Cultured SGNs from P3 WT mice were treated with 50 µM cisplatin for 48 h in the presence or absence of the ferroptosis activator erastin (25 µM) or the ferroptosis inhibitor DFO (80 µM). B-D) Erastin pretreatment significantly increased the expression of TfR1 (B) and the intensity of the FerroOrange signal (C) and aggravated the decrease in the TMRM signal intensity (D) in cultured SGNs treated with cisplatin, while DFO had the opposite effects. n = 4 for each group. B, C: scale bar = 25 µm. D: scale bar = 5 µm. DFO, deferoxamine.


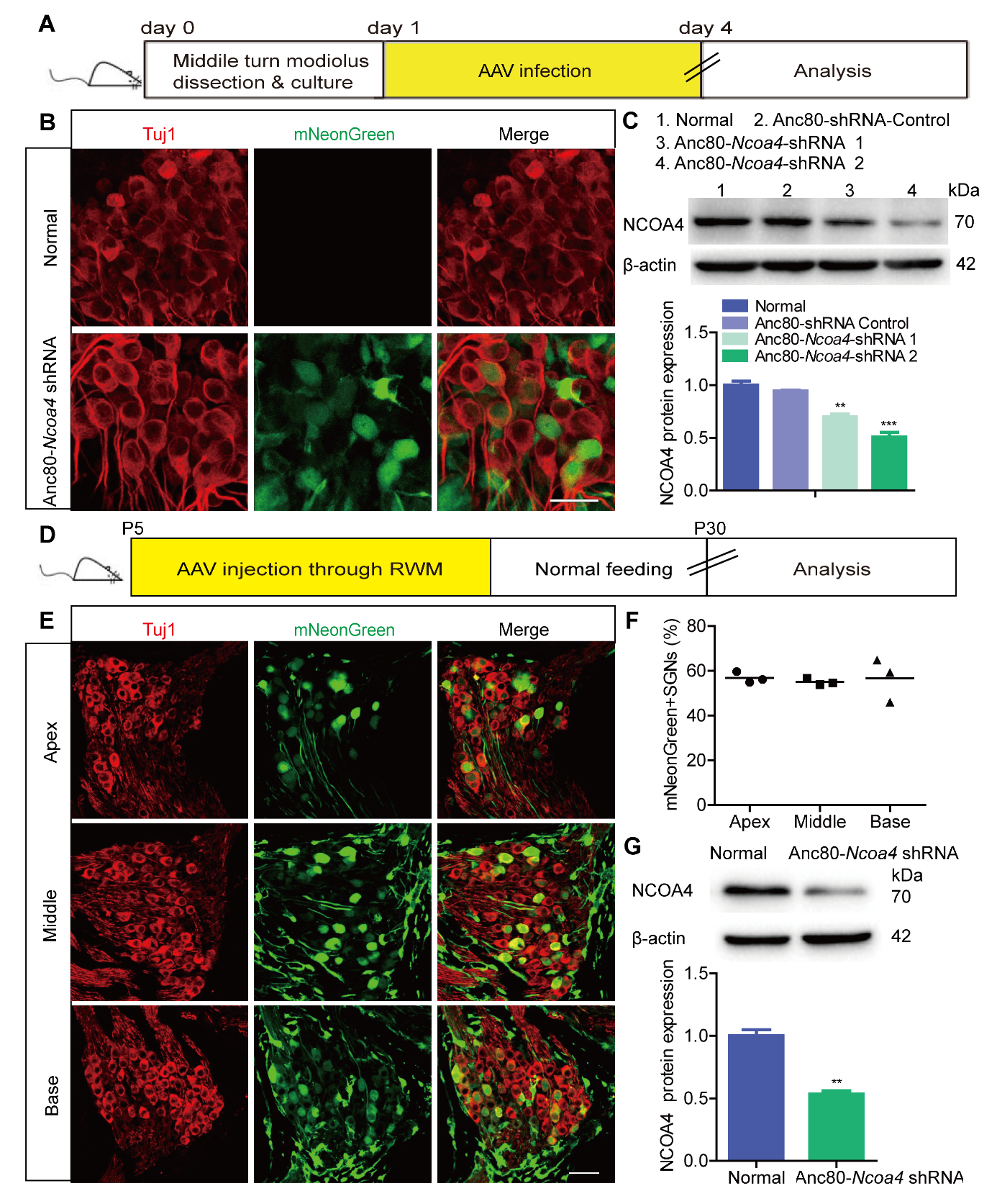


**Figure S2.** The transfection efficiency of Anc80-*Ncoa4* shRNA in cultured SGNs and in mice. A) Cultured SGNs from P3 WT mice were incubated with 4×10^10^ GC/mL Anc80-*Ncoa4* shRNA for 24 h, after which the medium was replaced with normal medium, and the SGNs were further incubated for 48 h. B, C) Approximately 60% of the cultured SGNs were successfully transfected with Anc80-*Ncoa4* shRNA, and Anc80-*Ncoa4* shRNA 2 was selected for subsequent experiments based on its relatively superior knockdown efficiency. n = 3 for each group. Scale bar = 25 µm. D) WT mice were injected with 1×10^10^ GCs of Anc80-*Ncoa4* shRNA via the RWM at P5, and the mice were sacrificed at P30. E-G) Approximately 60% of the SGNs in the apical, middle, and basal turns expressed mNeonGreen fluorescence, and the expression of NCOA4 was significantly reduced in the inner ear of P30 mice. n = 3 for each group. Scale bar = 25 µm. The data are presented as the mean ± S.D. ** *p* < 0.01, *** *p* < 0.001, two-tailed, unpaired Student’s t-tests.


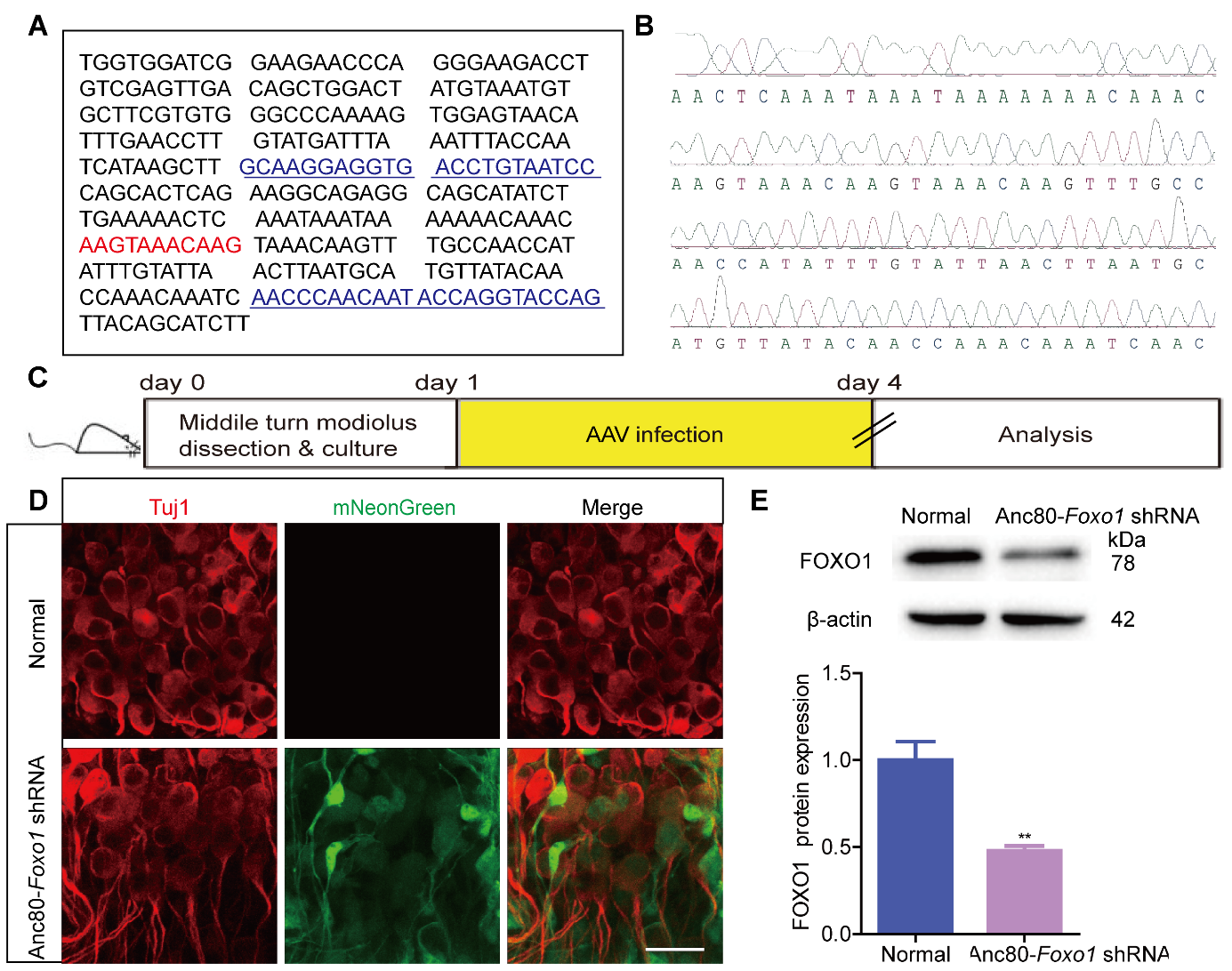


**Figure S3.** Sequencing of the PCR products obtained in the ChIP assay and the transfection efficiency of Anc80-*Foxo1* shRNA in cultured SGNs. A) The mouse NCOA4 promoter and primer sequences for the ChIP assay (underlined in blue), the potential binding site sequences of FOXO1 in NCOA4 promoter (red). B) The sequencing data from the ChIP‒PCR product provided further evidence of FOXO1 occupancy on the NCOA4 promoter. C) Cultured SGNs from P3 WT mice were incubated with 4×10^10^ GC/mL Anc80-*Foxo1* shRNA for 24 h, after which the medium was replaced with normal medium, and the SGNs were further incubated for 48 h. D, E) After transfection with Anc80-*Foxo1* shRNA, the expression of FOXO1 was effectively decreased in cultured SGNs. n = 3 for each group. Scale bar = 25 µm. The data are presented as the mean ± S.D. ** *p* < 0.01, two-tailed, unpaired Student’s t-tests.


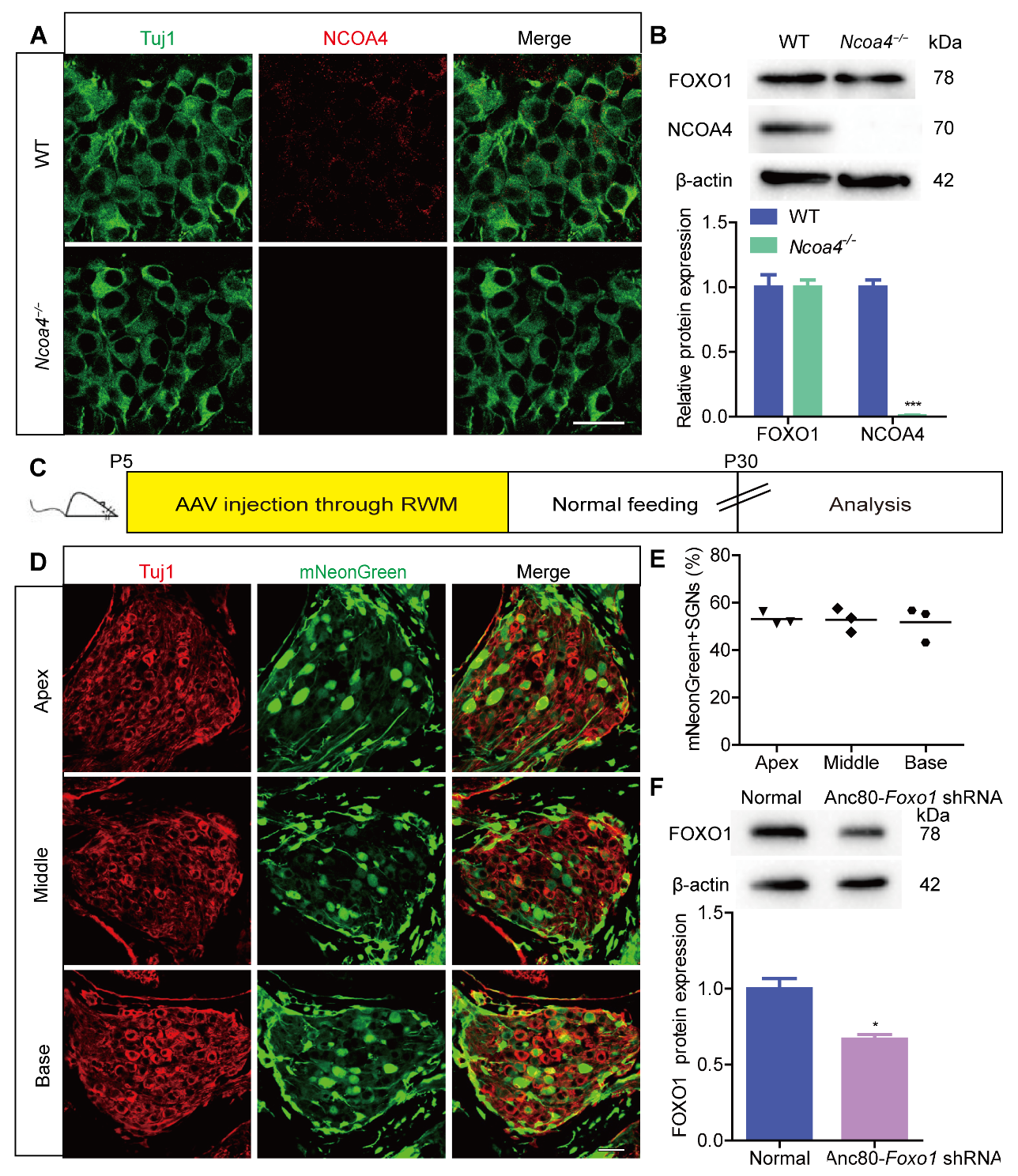


**Figure S4.** The expressions of FOXO1 and NCOA4 in cochlea and the transfection efficiency of Anc80-*Foxo1* shRNA in SGNs in mice. A, B) NCOA4 was expressed in cochlea SGNs while the expression of NCOA4 was almost undetectable in SGNs of *Ncoa4*^−/−^ mice. No change of FOXO1 expression was observed in *Ncoa4*^−/−^ mice cochlea. n = 3 for each group. Scale bar = 25 µm. C) The mice were injected with 1×10^10^ GCs of Anc80-*Foxo1* shRNA *via* the RWM at P5 and were sacrificed at P30. D-F) Approximately 52% of the SGNs in the apical, middle, and basal turns expressed mNeonGreen fluorescence. The expression of FOXO1 was significantly reduced in the P30 mice cochlea. n = 3 for each group. Scale bar = 25 µm. The data are presented as the mean ± S.D. * *p* < 0.05, ** *p* < 0.01, two-tailed, unpaired Student’s t-tests.

Supporting Information 2

**FOXO1-NCOA4 Axis Contributes to Cisplatin-Induced Cochlea Spiral Ganglion Neuron Ferroptosis via Ferritinophagy**

*Xue Wang^#^, Lei Xu^#^, Yu Meng^#^, Fang Chen, Jinzhu Zhuang, Man Wang, Weibin An, Yuechen Han, Bo Chu, Renjie Chai^*^, Wenwen Liu^*^, Haibo Wang^*^*

Original images of gel blots

Supporting Data 1: Raw gel blots showing Western blot analysis of NCOA4 protein expression. Protein samples were separated by SDS-PAGE and immunoblotted with anti-NCOA4 antibody. The band corresponds to the expected molecular weight of NCOA4. This image represents the original data used to generate the results presented in Figure 5B.

Cis+ Anc80-*Ncoa4* shRNA

Control

Marker

Marker

Cisplatin


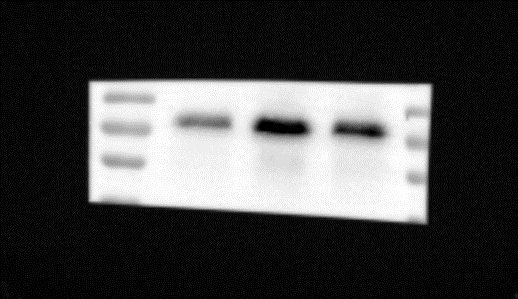


NCOA4 70 kDa

Supporting Data 2: Western blot analysis of FOXO1 and β-actin respectively. These images represent the original data used to generate the results presented in Supplementary Figure 3E.

Anc80-*Foxo1* shRNA

Marker

Marker

Normal


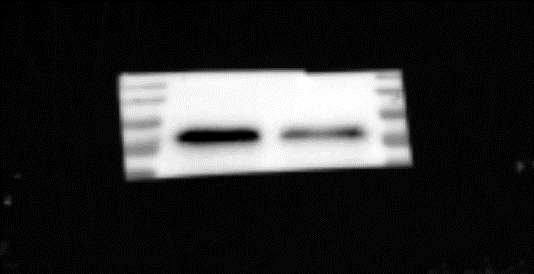


FOXO1 78 kDa


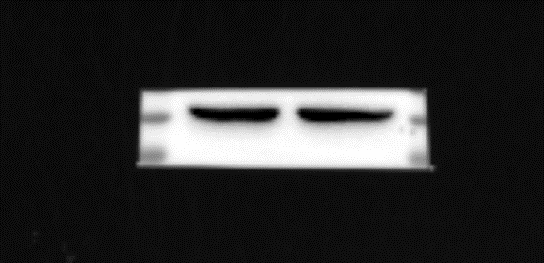


β-actin 42 kDa
